# Cardiac-specific ablation of the E3 ubiquitin ligase Mdm2 leads to oxidative stress, broad mitochondrial deficiency and early death

**DOI:** 10.1371/journal.pone.0189861

**Published:** 2017-12-21

**Authors:** Ludger Hauck, Shanna Stanley-Hasnain, Amelia Fung, Daniela Grothe, Vivek Rao, Tak W. Mak, Filio Billia

**Affiliations:** 1 Toronto General Research Institute, Toronto, Ontario, Canada; 2 Division of Cardiovascular Surgery, UHN, Toronto, Ontario, Canada; 3 Campbell Family Cancer Research Institute, Princess Margaret Hospital, Toronto, Ontario, Canada; 4 Division of Cardiology, University Health Network (UHN), Toronto, Ontario, Canada; 5 Heart and Stroke Richard Lewar Centre of Excellence, University of Toronto, Toronto, Ontario, Canada; 6 Institute of Medical Science, University of Toronto, Toronto, Ontario Canada; Rutgers New Jersey Medical School, UNITED STATES

## Abstract

The maintenance of normal heart function requires proper control of protein turnover. The ubiquitin-proteasome system is a principal regulator of protein degradation. Mdm2 is the main E3 ubiquitin ligase for p53 in mitotic cells thereby regulating cellular growth, DNA repair, oxidative stress and apoptosis. However, which of these Mdm2-related activities are preserved in differentiated cardiomyocytes has yet to be determined. We sought to elucidate the role of Mdm2 in the control of normal heart function. We observed markedly reduced Mdm2 mRNA levels accompanied by highly elevated p53 protein expression in the hearts of wild type mice subjected to myocardial infarction or trans-aortic banding. Accordingly, we generated conditional cardiac-specific Mdm2 gene knockout (*Mdm2*^f/f^;*mcm*) mice. In adulthood, *Mdm2*^f/f^;*mcm* mice developed spontaneous cardiac hypertrophy, left ventricular dysfunction with early mortality post-tamoxifen. A decreased polyubiquitination of myocardial p53 was observed, leading to its stabilization and activation, in the absence of acute stress. In addition, transcriptomic analysis of Mdm2-deficient hearts revealed that there is an induction of E2f1 and c-Myc mRNA levels with reduced expression of the Pgc-1a/Ppara/Esrrb/g axis and Pink1. This was associated with a significant degree of cardiomyocyte apoptosis, and an inhibition of redox homeostasis and mitochondrial bioenergetics. All these processes are early, Mdm2-associated events and contribute to the development of pathological hypertrophy. Our genetic and biochemical data support a role for Mdm2 in cardiac growth control through the regulation of p53, the Pgc-1 family of transcriptional coactivators and the pivotal antioxidant Pink1.

## Introduction

Heart failure occurs when the heart is unable to adequately pump blood to meet the demands of the body at normal filling pressures. It is a clinical syndrome that is defined by symptoms of fatigue, shortness of breath and fluid retention [1–5] and is the leading cause of morbidity and mortality in North America. Heart failure has been identified as the second leading cause of extended hospital stays rendering this disease an ever-increasing socioeconomic burden [6–8]. The quality of life and the prognosis for this group of patients remains poor with one-year survival rates less than 40% [9].

In mammals, adult cardiomyocytes are terminally-differentiated cells with a rather limited capacity to proliferate to any appreciable extent for cardiac regeneration after injury [10–14]. This renders the heart particularly vulnerable to ischemic, toxic or biomechanical injury. To compensate for the irreversible damage, the left ventricle undergoes hypertrophy and remodeling. In mice, hypertrophic growth of cardiomyocytes is a result in reprogramming of fetal genes [15–17]. Although this response is considered initially compensatory, cardiomyocyte hypertrophy eventually leads to alterations in myocyte gene expression, metabolism, excitation contraction coupling, accompanied with enhanced apoptosis and fibrosis which is ultimately detrimental and progressive left ventricular dysfunction develops [18–22].

Cardiac remodeling is associated with protein synthesis and degradation. While elevated protein synthesis is a well-established process in cardiac hypertrophy, the link between proteasome-mediated protein degradation and cardiac hypertrophy is less well understood [23–26]. The Ubiquitin-Proteasome System (UPS) represents the major protein degradation pathway regulating a multitude of signaling pathways [27]. However, while inhibition of proteasomal function has proven beneficial towards maintaining cardiac homeostasis *in vivo*, this approach has suffered poor clinical translation [28–30]. Currently there is a great impetus to utilize downstream specificity of the UPS via the E3 ubiquitin ligases that ubiquitinate specific substrate proteins leading to their degradation. Indeed, harnessing E3 ubiquitin ligase specificity to regulate key factors governing cardiac homeostasis and hypertrophy holds potential for therapeutic intervention.

The E3 ubiquitin ligase Mdm2 is ubiquitously expressed as a multi-domain member of the RING finger domain family that attaches mono- and/or poly-ubiquitin chains onto its target substrates [31]. Mdm2 targets many proteins regulating pleiotropic biological processes including cell growth, proliferation and apoptosis, such as the tumor suppressors Trp53 (p53), retinoblastoma (*Rb1*) or the transcription factor E2f1 and targets them for proteasomal degradation [32–34].

The p53/Mdm2 pathway is one of the best characterized circuitries [35,36] that translates growth and survival signals into specific gene expression patterns, regulating tumor-free survival of an organism [37–39]. In normal cells, p53 expression is kept at low levels by Mdm2, through proteasomal degradation. In response to acute stress, Mdm2 is inactivated and increased p53 levels block cell division and induce apoptosis. Conversely, p53 can activate Mdm2 transcription, thereby forming a negative feedback loop that curtails p53 activity. Mdm2 is required for regulation of p53 *in vivo* since Mdm2 knockout mice die early in embryogenesis due to p53-dependent apoptosis [40].

Regulation of growth and apoptosis in mitotic cells and tissues by the p53/Mdm2 circuitry has been well established [41–43]. Intriguingly, Akt-dependent phosphorylation of Mdm2 increases its ubiquitination activity which results in downregulation of p53 thereby enhancing cellular survival and proliferation [44]. However, apart from the analysis of Mdm2 in transgenic mice or germline knockout models [45–47], the precise physiological function of Mdm2 in the adult heart has yet to be determined. Therefore, we recognized the importance of investigating the potential role of Mdm2 as a regulator of growth regulated processes.

In this study, we report significant decreases in Mdm2 mRNA levels in samples obtained from patients with end-stage heart failure. We also describe Mdm2 and p53 transcript and protein expression levels in murine models of heart failure, and evaluate the alterations in cardiac function, energy metabolism and reactive oxygen species (ROS) defense in the presence or absence of Mdm2 in the adult mouse heart. We show that p53 is a specific Mdm2 target and that ablation of Mdm2 leads to activation of p53 in the absence of acute stress. The loss of Mdm2 severely impacts redox homeostasis and mitochondrial function through activation of the transcription factors c-Myc and E2f1, both known for their ability to induce apoptosis and inhibit antioxidative systems. Under these conditions, negative regulation of the Pgc-1a/Ppar/Esrr axis and Pink1 evoked oxidative stress, energy deprivation and cardiac dysfunction. All these processes are Mdm2-dependent events, and can contribute to the development of pathological hypertrophy, left ventricular dysfunction and premature mortality. Our data identify Mdm2 as a regulator of cardiac homeostasis through the persistent upregulation of ROS-detoxifying systems and mitochondrial energy metabolism.

## Materials and methods

### Cardiac-specific *Mdm2*^f/f^;*mcm* conditional mutant mice

To determine if Tam and Cre expression is toxic to cardiomyocytes we included vehicle- and Tam-injected wild type C57BL/6J, MerCreMer (*mcm*) and *Mdm2*^f/f^ mice in all initial analyses of the corresponding mutants. We found that mice of these experimental groups were phenotypically indistinguishable from vehicle-injected *Mdm2*^f/f^;*mcm* control animals used in this study, as judged by heart body weight ratios, ventricular fibrosis and fractional shortening. All animal usage in this study was in accordance with approved institutional animal care guidelines of the UHN (AUP 1815/1379, Canadian Council in Animal Care). All animals used in this study were 10 weeks old (22-26g) at the beginning of experimentation. All experiments used isogenic controls of matched age and sex.

The *Mdm2*^f/f^ (01XH9) and *p53*^f/f^ mice (strain number 01XC2) mice were obtained from Frederick (Rockville, MD 20852 USA). These strains were previously backcrossed into a C57BL/6J background for 7 generations. The *mcm* strain (Tg(Myh6-cre/Esr1*)1Jmk/J) was from Jackson (Bar Harbor, ME 04609 USA). In these mice, the cardiac muscle a-myosin heavy chain 6 promoter drives the expression of Cre (c) recombinase fused to two mutant (*m*) estrogen-receptor ligand-binding domains (*mcm*) when exposed to Tam [48]. We crossed *mcm* transgenic mice on a C57BL/6J background with mice carrying the conditional alleles *Mdm2*^f/f^ to obtain *Mdm2*^f/f^;*mcm* animals. We crossed *mcm* transgenic mice on a C57BL/6J background with mice carrying the conditional alleles *p53*^f/f^ to obtain *p53*^f/f^;*mcm* animals. We crossed the heterozygous offspring of *p53*^f/f^;*mcm* and *Mdm2*^f/f^;*mcm* breeders to obtain *Mdm2*^f/f^;*p53*^f/f^;*mcm* animals. The *Mdm2*^f/+^;*mcm* strain was obtained by crossing *Mdm2*^f/f^;*mcm* mice with wild type (C57BL/6J) animals. We obtained *Mdm2*^f/f^;*p53*^f/+^;*mcm* mice by breeding *Mdm2*^f/f^;*p53*^f/f^;*mcm* animals with *Mdm2*^f/f^;*mcm* mice. After weaning, experimental male mice were housed in groups of 3–5 animals in mechanically ventilated cages (600 cm^2^; changed fortnightly) environmentally enriched by bedding and nesting materials and crawl tubes. Animals were held in a temperature-controlled environment at 19–22°C on a diurnal 12h light cycle. Mice were provided free access to standard non-medicated pelleted laboratory rodent chow (Harlan) and tap water ad libitum from a portable water faucet.

DNA isolated from fresh tail snips was employed for genotyping. Samples were incubated in 300ul of 50 mM NaOH for 2h, 80°C while rocking. Then, samples were neutralized with 25 ul 1.0 M HCl, 700 ul H_2_O, vigorously vortexed, centrifuged for 13,000 rpm for 10 min and stored at 4°C. We used 0.5 ul of DNA solution per PCR reaction and the following primers: Mdm2 forward 5’-CTGTGTGAGCTGAGGGAGATGTG-3’; Mdm2 reverse 5’-CCTGGATTTAATCTGCAGC ACTC-3’. p53 forward 5’-CACAAAAACAGGTTAAACCCAG-3’; p53 reverse 5’-AGCACAT AGGAGGCAGAGAC-3’. Mcm forward 5’-AGGTGGACCTGATCATGGAG-3’; mcm reverse 5’-ATACCGGAGATCATGCAAGC-3. We performed PCR analysis with Quanta Accustart Geltrac with GelDye (no. 95136-04K; VWR) and Platinum Blue Supermix (no. 12580–023; Invitrogen): Mdm2 wild-type allele, 310 bp; Mdm2 floxed allele, 397 bp; mcm 440 bp. p53 wild type allele, 288 bp. p53 floxed allele 370 bp. Genomic recombination efficiency at the *Mdm2* gene locus was measured by PCR employing the following primers spanning exon 7 to 9: forward 5’-CCTCCCCCACGTCTACTAAA-3’; reverse 5’-TGTTTTGACAGCAGGTCAGG -3’. Amplicon size: 590 bp.

An ethanol-peanut oil emulsion of 4-Hydroxytamoxifen (Tam; H6278, Sigma-Aldrich) was prepared by completely dissolving 100 mg Tam in 5 ml highly purified ethanol (ACS reagent grade, anhydrous, absolute; no. 6590–32; Ricca Chemical, Fisher) while vortexing vigorously for 5–8 min. Peanut oil (32 ml) (P2144; Sigma-Aldrich) was added and the emulsion was again vigorously vortexed for 2 min. Then, the emulsion was sonicated on ice at highest output for 10–30 sec until it became translucent, aliquoted, and stored at -20°C for several months. Shortly before usage, Tam was melted in a 37°C water bath, briefly vortexed, and 200–250 ul were immediately injected intraperitoneally into conscious mice. Animals were injected daily between 5–6 pm for four consecutive days (98–83 ug/g body weight cumulative dosage). Homologous recombination was completed at 7 days post-Tam. Vehicle control mice were intraperitoneally injected with an ethanol-peanut oil emulsion lacking Tam.

For 5-bromo-2´-deoxyuridine (BrdU) (B5002; Sigma-Aldrich) labeling *in vivo*, BrdU was dissolved in PBS (10 mg/ml), aliquoted and stored at -20°C. Conscious mice were intraperitoneally injected with 200 ul BrdU (cumulative dosage of 160 mg/kg) twice daily at 6d and 7d post-Tam, and sacrificed at 16 hours after the last injection. The membrane permeable proteasome inhibitor MG132 as ready-made solution in DMSO was purchased from Sigma (M7449). At 7 days post-Tam, mice were injected with MG132 (30 umol/kg body weight) and sacrificed 6 hours later [49,50].

### Microarray analysis

All microarray data were submitted to the ArrayExpress database (Experiment ArrayExpress accession: E-MTAB-5441; http://www.ebi.ac.uk/arrayexpress).

Total RNA from mouse LV tissues was isolated with Trizol reagent (no. 15596026; Thermo Fisher Scientific). Phase lock Gels (no. 826754; VWR) were employed to eliminate interphase-protein contaminations. RNA quality was assessed by 260/280 and 260/230 absorption ratios employing a Nanodrop spectrophotometer (NanoDrop; Thermo Fisher Scientific), and an Agilent Bioanalyzer at the Microarray Facility, Centre for Applied Genomics, The Hospital for Sick Children (Toronto). RNA samples were processed for analysis by Affymetrix Mouse Gene 1.0 ST expression arrays at the Centre for Applied Genomics. Processing of probe level data and all subsequent analyses were performed using GeneSpring (Version 13.2; Agilent Technologies Inc.). Genome wide data from the gene expression microarrays were normalized and filtered for genes with significant expression levels (log_2_ fold change ±1.3; *P* < 0.05) in Tam-treated *Mdm2*^f/f^;*mcm* compared with vehicle injected control *Mdm2*^f/f^;*mcm* (*n* = 3) employing GeneSpring. Selection of top-ranked differentially expressed genes in the GO gene sets was performed on the bases of arbitrary threshold for fold changes plus statistical significance according to the *t* test with Benjamini-Hochberg correction (log_2_ fold change ±1.3; *P* < 0.01).

### Statistical analyses

Data are means±s.e.m. as indicated at the bottom of each Figure legend. We used factorial design analysis of variance (ANOVA) or t-tests to analyze data as appropriate employing GraphPad InStat (version 3.1) and GraphPad Prism (GraphPad Software, version 7.0; La Jolla, CA 92037 USA). Significant ANOVA values were subsequently subjected to simple main effects analyses or *post hoc* comparisons of individual means using the Tukey method as appropriate. We considered *P* values of < 0.05 as significant.

### Myocardial infarction, transaortic banding and echocardiography

For myocardial infarction, mice were subjected to ligation of the left anterior descending artery at the level of the left atrial appendage or sham operation. For transaortic banding (TAB), mice were anesthetized using 2% (vol/vol) isoflurane/95% (vol/vol) oxygen and ventilated. A left thoracotomy was performed and the pericardium opened. The transverse aortic arch was ligated using a 30-gauge needle with an overlying suture. Sham mice underwent a comparable operation in which a suture was passed without ligation.

Echocardiography on anesthetized mice (2.0% isoflurane, 98% oxygen) was performed using a 15-MHz linear ultrasound transducer (Vivid7; GE). Body temperature was maintained at 37°C. M-mode measurements of the LV end-diastolic diameter (LVEDD) and LV end-systolic diameter (LVESD) were made in triplicate from short-axis views at the level of the papillary muscle and averaged over three to six beats. LVEDD was measured at the time of the apparent maximal LV diastolic dimension, whereas LVESD was measured at the time of the most anterior systolic excursion of the posterior wall. LV fractional shortening (FS) was calculated as follows: FS = (LVEDD − LVESD)/LVEDD × 100%.

### Immunofluorescence microscopy, morphometric analyses, apoptosis and DNA replication assays

Mice were euthanized without anesthesia by cervical dislocation between 9-11am. Anticoagulant was not administered. Hearts were quickly excised and rinsed in 20 ml ice-cold PBS, pH 7.4 without Ca^2+^/Mg^2+^ (no. 10010023; Thermo Fisher Scientific), fixed in 4% PBS-buffered formalin (10 ml) for 50 min at room temperature with constant agitation and incubated in 0.3 M glycine in PBS (pH 7.4; 10 ml) at 4°C for 3 to 5 days. After embedding hearts in Tissue-Tek OCT Compound (Sakura, Finetek; VWR) sections were cut at 10 um thickness using a cryostat (HM525 NX; Thermo Fisher Scientific), and mounted on histological slides (Superfrost Plus Microslides; no. 48311–703; VWR). For BrdU (5-Bromo-2′-deoxyuridine) labeling experiments, DNA was denatured in 2N HCl for 30 min post-fixation. Specimen were then permeabilized and stained with antibodies to cardiac-specific nuclear Mef2a and BrdU. Detection of fragmented genomic DNA was performed by terminal deoxynucleotidyl transferase-mediated dUTP nick-end-labeling (TUNEL) according to the manufacturer’s instructions (no. 1684795910; Roche). These samples were co-stained with cardiac-specific anti-sarcomeric a-actinin after permeabilization. Specimen were permeabilized in 1.0% Triton X-100 (X100; Sigma-Aldrich) in TBS (20 mM Tris, 150 mM NaCl), pH 7.6 for 60 minutes at room temperature. Samples were incubated with primary antibodies (listed in Table 1 in TBS/1.0% Triton X-100, for 16–20 hours at room temperature without agitation. Thereafter, specimen were briefly rinsed with TBS. For visualization, samples were incubated with secondary antibodies, diluted 150-fold in TBS/1.0% Triton X-100 for 45 min in the dark (Alexa Fluor 488-goat anti-rat IgG, A-11006; Alexa Fluor 555-goat anti-rabbit IgG, A-21429; Alexa Fluor 555-goat anti-mouse IgG, A-2142; Alexa Fluor 647-goat anti-mouse IgG, A-20990 (Thermo Fisher Scientific). Specimen were briefly rinsed with TBS, and nuclear DNA was visualized with 4,6-diamidino-2-phenylindole (Dapi; 1.0 ug/ml in PBS) (D9542; Sigma-Aldrich). ProLong Diamond antifade reagent (no. 36965; Thermo Fisher Scientific) was applied, and samples were sealed with a coverslip. Three-dimensional confocal laser scanning microscopy was performed on a Zeiss LSM700 confocal microscope and LSM Zen 2009 data acquisition software (AOMF-Advanced Optical Microcopy Facility, Ontario Cancer Institute, Toronto, ON Canada). For determination of cardiomyocyte cell size and ventricular remodeling, ventricular samples were stained with sarcomeric a-actinin and Alexa Fluor 488-conjugated wheat germ agglutinin (WGA) (W11261; Thermo Fisher Scientific). Cross-dimensions of adult cardiomyocytes and fibrotic area were determined by planimetry of immunofluorescence microphotographs using ImageJ (Version 1.51d; National Institutes of Health, Bethesda, MD, https://imagej.nih.gov/ij/). After recording, simple adjustments and assembly of entire and cropped microphotographs were performed employing Adobe Photoshop CS6.

**Table 1:** Antibodies employed for IF, IP and WB^a^.

Gene name	Catalog No.	Vendor	Application	Dilution Factor
**Aco2**	6922	Cell Signaling	WB	1000
**a-actinin, sarcomeric**	A7811	Sigma	IF	50
**Atp5a**	ab14748	Abcam	WB	1000
**Bak**	3184	Cell Signaling	WB	1000
**Bax**	2772	Cell Signaling	WB	1000
**BrdU**	MCA2060	AbD Serotec	IF	50
**Cat**	14097	Cell Signaling	WB	1000
**Complex I**	ab109798	Abcam	ELISA	5 ug
**Cox4i1**	ab16056	Abcam	WB	1000
**Cx43**	CBL171	Sigma	WB	1000
**CytC**	ab13575	Abcam	WB	1000
**E2f1**	sc-193	Santa Cruz	WB	500
**Erk1/2**	9102	Cell Signaling	WB	1000
**Erk1/2 Pi-Thr202/Tyr204**	9101	Cell Signaling	WB	1000
**Esrra/b**	NBP1-47254	Novus	WB	1000
**Esrrg**	27–388	ProScience	WB	1000
**Gpx4**	Ab125066	Abcam	WB	1000
**Gsta1**	sc-100546	Santa Cruz	WB	500
**Lam**	L9393	Sigma	WB	1000
**Mdm2**	M8558	Sigma	WB	1000
**Mef2a**	ab32866	Abcam	IF	50
**Myc**	sc-764	Santa Cruz	WB	500
**Ndufa8**	BS3336	BioWorld	WB	1000
**normal rabbit IgG**	2729	Cell Signaling	IP	7.5 ug—1000
**Npm1**	B0556	Sigma	WB	1000
**Opa1**	ab42364	Abcam	WB	1000
**p38**	9112	Cell Signaling	WB	1000
**p38 Pi-Thr180/Tyr182**	9211	Cell Signaling	WB	1000
**p53**	BML-SA293	Enzo Life Sci.	IP WB	1000
**Pgc—1a**	3934–100	BioVision	WB	1000
**Pink1**	3929–100	BioVision	WB	1000
**Pln**	8495	Cell Signaling	WB	1000
**Ppara**	PAB11321	Abnova	WB	1000
**Sdhb**	ab14714	Abcam	WB	1000
**SERCA2**	9580	Cell Signaling	WB	1000
**Sirt3**	5490	Cell Signaling	WB	1000
**Sod2**	611580	Becton Dickinson	WB	4000
**Txnrd2**	ab16841	Abcam	WB	1000
**ubiquitin**	13–1600	Thermo Fisher	WB	7.5 ug

^a^IF, immunofluorescence microscopy. IP, immunoprecipitation. WB, Western blot.

### Cell fractionation, total heart tissue extracts and isolation of mitochondria

Subcellular cell fractions were prepared using the NE-PER Kit (no. 78833; Pierce) supplemented with phosphatase inhibitors (1.0 mM Na_3_VO4, 20 mM NaF, 10 mM b-glycerophosphate; Sigma) and with a protease inhibitor cocktail containing AEBSF, Aprotinin, Bestatin, E64, Leupeptin and Pepstatin (no. P2714; Sigma). Ventricular specimen were mechanically homogenized in RIPA Buffer (no. 9806; Cell Signaling) composed of 20 mM Tris-HCl (pH 7.5), 150 mM NaCl, 1.0 mM EGTA, 1.0% NP-40, 1.0% sodium deoxy cholate, 2.5 mM sodium pyrophosphate, 1.0 mM b-glycerophosphate, 1.0 mM Na_3_VO_4_, 1.0 mg/ml leupeptin, phosphatase and protease inhibitors. Mechanochemical assisted isolation of mitochondria from adult mouse ventricular tissues was performed using extraction buffer A (10 mM HEPES, pH 7.5; 200 mM mannitol; 70 mM sucrose; 1.0 mM EGTA) supplied with the Mitochondrial Isolation Kit (MITOISO1; Sigma) according to the manufacturer’s instructions. Isolated mitochondria derived from one heart were resuspended in 200 ul storage buffer (10 mM HEPES, pH 7.5, containing 250 mM sucrose, 1.0 mM ATP, 80 uM ADP, 5.0 mM sodium succinate, 2.0 mM K_2_HPO_4_, 1.0 mM DTT) (MITOISO1; Sigma), and used directly in mitochondrial complex activity assays, or aliquoted and stored at -80°C for Western blot analysis. The protein concentration was approximately 3.0–4.0 ug/ul as determined by fluorometry (Qubit 2.0 Fluorometer; no. Q33217; Thermo Fisher Scientific).

### Mitochondrial Complex I function

The relative activity of mitochondrial Complex I was determined employing the Complex I Enzyme Activity Assay kit (MS141; Abcam), according to the manufacturer’s procedures except that the supplied 96-well plates were re-coated with antibodies to Complex I or IV (5.0 ug/well) and freshly purified mitochondria were used (50 ul/well). The Complex I kit determines the diaphorase-type activity of Complex I. This activity is not dependent on the presence of ubiquinone and, therefore, inhibitors, such as rotenone, do not inhibit Complex I activity in this assay. Importantly, the activity assay is affected by assembly deficiencies, and, thus enabled us to specifically determine the impact of decreased Ndufa8 levels on Complex I activity. Colorimetric increases in absorbance at 450 nm for Complex I assays were recorded at room temperature for 30 min at 2 min intervals using a spectrophotometer (FlexStation 3; Molecular Devices).

### Immunoprecipitation assays and immunoblotting

For immunoprecipitation (IP) assays, cellular extracts in supplemented RIPA Buffer were incubated with antibodies to ubiquitin (7.5 ug) or normal rabbit IgG (7.5 ug) covalently linked to protein A agarose beads (Seize X Protein A IP Kit; no. 26149; Pierce) for 3 hours at 4°C. Immunocomplexes were washed twice with lysis buffer and boiled in SDS sample buffer (no. 7722; Cell Signaling). Protein samples were resolved by SDS-PAGE employing 4–12% and 3–8% NuPAGE pre-cast gels (Life Technologies), and PVDF membranes (iBlot; Life Technologies). The presence of ubiquitinated p53 in the immunoprecipitate was determined by immunoblotting with anti-ubiquitin antibodies. The following secondary antibodies were employed for chemiluminescence detection of proteins: horseradish peroxidase (HRP)-conjugated anti-rabbit IgG (no. 7074; Cell Signaling), HRP-conjugated anti-mouse IgG (no. 7076; Cell Signaling), and Luminata Crescendo (WBLUR0100, Millipore). The complete list of antibodies employed in our study can be found in Table 1.

### Detection of oxidative damage, antioxidants levels, and metabolism assays

Individual ventricular specimen were rapidly cut into 4–6 pieces, briefly rinsed in 40 ml ice-cold PBS, immediately snap frozen in liquid nitrogen, and stored at -80°C. Assays for determination of small metabolic molecules (4-HAE, ATP, GSH/GSSG) were deproteinized prior to analysis using the perchlorate-based precipitation based Deproteinizing Sample Preparation Kit according to the supplier’s instruction (no. K808-200; BioVision). All assays were performed according to the manufacturer’s instructions with minor modifications: 4-HAE/MDA (Bioxytech LPO-586 kit; Oxis), 8-OHdG (Bioxytech 8-OHdG-EIA kit; Oxis), ATP (ENLITEN ATP Assay System Bioluminescence no. FF200; Promega), ADP (ADP-Glo Kinase Assay V6930; Promega), aconitase (Bioxytech Aconitase-340 kit; Oxis), caspase-3 and 7 (Caspase-Glo 3/7 Assay; G8090; Promega), caspase 8 (Caspase-Glo 8 Assay; G8200; Promega) catalase (Bioxytech Catalase-520 kit; Oxis), and GSH/GSSG (Bioxytech GSH/GSSG-412 kit; Oxis). Each sample was measured in duplicate. Final values were normalized by the total protein concentration for each sample determined prior to precipitation.

### Determination of the mitochondrial membrane potential (Δ*Ψm*)

The membrane permeable fluorescence dye JC-1 (no. T3168; Thermo Fisher Scientific) is a mitochondria membrane potential (MMP) probe that exhibits potential-dependent accumulation in mitochondria, indicated by a fluorescence emission shift from green (525 nm) to red (590 nm). This depolarization of MMP occurs at early stages of oxidative stress and cell death. This potential-sensitive emission shift from green to red is due to concentration-dependent formation of red fluorescent J aggregates, which in turn is dependent on MMP. Thus, decreases in MMP are measured by decreases in the intensity of emitted red fluorescence. Isolated mitochondria (10 ul/well) in HBSS (without Ca^2+^/Mg^2+^, phenol red no.14185-052; Thermo Fisher Scientific) were incubated with JC-1 (5.0 ug/ml in NADH-containing Complex I assay buffer; MS141, Abcam) for 1–2 min and then treated with antimycin. JC-1 emission at 525/595 nm was recorded (2 readings/min for 30 min) using a fluorescence spectrophotometer (Flex Station 3; Molecular Devices). The rate between two time points (emission at 595 nm/min) was calculated in the most linear range of decline for JC-1 emission. ROS levels were determined employing the 2’,7’-dichlorofluorescin diacetate (DCFDA) Cellular Reactive Oxygen Species Detection Assay Kit (ab113851; Abcam) according to the manufacturer’s instructions.

### Mitochondrial biogenesis and capacity

Impaired mitochondrial (mt) biogenesis is a key marker of physiologic stress or genetic mutations. Mt have multiple circular genomes (mtDNA) that are replicated independently from the nuclear genome (nDNA). The mtDNA encodes 13 core polypeptides involved in oxidative phosphorylation in addition to 22 tRNA and 12S and 16S rRNA genes for mt protein synthesis. We used the relative mtDNA copy as a surrogate for mitochondrial biogenesis. Therefore, the mtDNA copy number was determined by qPCR of the mitochondrial gene cytochrome b (mtCytb) normalized to levels of a nuclear (n) encoded single-copy gene, B2m, using DNA preparations from total ventricular lysates. The ratio of mtDNA to nDNA in ventricle-injected hearts was arbitrarily set to 1. We used mitochondrial gene transcription as a surrogate for mitochondrial capacity. Therefore, the mRNA expression levels of the mtCytb gene were measured by RT-qPCR and corrected for the transcript expression of nuclear B2m. The ratio of transcript levels of mtCytb to B2m in ventricle-injected hearts was set to 1.

### Reverse transcription and quantitative real time PCR assays

We carried out two-step reverse transcriptase (RT) and quantitative real-time polymerase chain reactions (qPCR) on a LightCycler 480 (Roche; TMDT Core Facility) for mRNA analysis. Total RNA from mouse and human ventricular cardiac specimen was isolated with Trizol reagent (no. 15596026; Thermo Fisher Scientific), and Phase-lock Gels (no. 826754; VWR) were employed to eliminate interphase-protein contaminations. We used 500 ng total RNA in a 20 ul reaction for first-strand cDNA synthesis employing the SensiFast cDNA synthesis kit (BIO-65053; Bioline). For qPCR, we employed 4.0 ul first-strand synthesis product, diluted 5-fold with MilliQ-grade water and the Quanta Accustart II PCR Supermix (no, 95136–04; VWR) with EvaGreen dye (no. 31000; VWR). Mouse qPCR primers [51] used in this study are summarized in Table 2. Relative quantification of transcript levels was performed using the ddCt method with normalization to beta-2 microglobulin (B2m) employing the data analysis module. Mdm2 transcript levels were determined in human samples with Taqman assays employing standard human gene probes for B2m (no. HS99999907_m1) and Mdm2 (HS01066930_m1; Thermo Fisher Scientific). Relative quantification of Mdm2 transcript levels was performed using the ddCt method with normalization to B2m employing the data analysis module.

**Table 2:** Primer sequences used in RT-qPCR assays.

Gene Symbol	Forward	Reverse
**Adra1a**	CTAAGGCCATTCTACTTGGGGT	CGAGTGCAGATGCCGATGA
**Adra1b**	CGGACGCCAACCAACTACTT	AACACAGGACATCAACCGCTG
**Adrb1**	CTCATCGTGGTGGGTAACGTG	ACACACAGCACATCTACCGAA
**Adrb2**	GGGAACGACAGCGACTTCTT	GCCAGGACGATAACCGACAT
**Ace2**	TCCAGACTCCGATCATCAAGC	GCTCATGGTGTTCAGAATTGTGT
**ANP**	GCTTCCAGGCCATATTGGAG	GGGGGCATGACCTCATCTT
**Apaf1**	AGTGGCAAGGACACAGATGG	GGCTTCCGCAGCTAACACA
**ATPaf1**	CCCCTTCTACGACCGCTAC	CCACTGGCTGCTTTCGGAA
**ATP5a1**	TCTCCATGCCTCTAACACTCG	CCAGGTCAACAGACGTGTCAG
**B2m**	TTCTGGTGCTTGTCTCACTGA	CAGTATGTTCGGCTTCCCATTC
**Bak1**	CAACCCCGAGATGGACAACTT	CGTAGCGCCGGTTAATATCAT
**Bax**	TGAAGACAGGGGCCTTTTTG	AATTCGCCGGAGACACTCG
**BNP**	GAGGTCACTCCTATCCTCTGG	GCCATTTCCTCCGACTTTTCTC
**Casp1**	ACAAGGCACGGGACCTATG	TCCCAGTCAGTCCTGGAAATG
**Casp3**	TGGTGATGAAGGGGTCATTTATG	TTCGGCTTTCCAGTCAGACTC
**Casp8**	TGCTTGGACTACATCCCACAC	TGCAGTCTAGGAAGTTGACCA
**Col1a1**	GCTCCTCTTAGGGGCCACT	CCACGTCTCACCATTGGGG
**Col1a2**	GTAACTTCGTGCCTAGCAACA	CCTTTGTCAGAATACTGAGCAGC
**Col4a1**	CTGGCACAAAAGGGACGAG	ACGTGGCCGAGAATTTCACC
**Col4a2**	GACCGAGTGCGGTTCAAAG	CGCAGGGCACATCCAACTT
**Col5a1**	CTTCGCCGCTACTCCTGTTC	CCCTGAGGGCAAATTGTGAAAA
**Col5a2**	TTGGAAACCTTCTCCATGTCAGA	TCCCCAGTGGGTGTTATAGGA
**Col6a1**	CTGCTGCTACAAGCCTGCT	CCCCATAAGGTTTCAGCCTCA
**Col6a2**	AAGGCCCCATTGGATTCCC	CTCCCTTCCGACCATCCGAT
**Col6a3**	GCTGCGGAATCACTTTGTGC	CACCTTGACACCTTTCTGGGT
**Col8a1**	ACTCTGTCAGACTCATTCAGGC	CAAAGGCATGTGAGGGACTTG
**Col11a1**	ACAAAACCCCTCGATAGAAGTGA	CTCAGGTGCATACTCATCAATGT
**Col12a1**	AAGTTGACCCACCTTCCGAC	GGTCCACTGTTATTCTGTAACCC
**Col15a1**	CCCAGGGAAGAATGGAGAAGT	CCAGAGCCTTCAATCTCAAATCC
**Cox4i1**	ATTGGCAAGAGAGCCATTTCTAC	CACGCCGATCAGCGTAAGT
**Cyc1**	CAGCTTCCATTGCGGACAC	GGCACTCACGGCAGAATGAA
**Cytb**	TTCTGAGGTGCCACAGTTATT	GAAGGAAAGGTATTAGGGCTAAA
**E2f1**	CTCGACTCCTCGCAGATCG	GATCCAGCCTCCGTTTCACC
**Esrra**	GCACCTGGGCTCTAGTTGC	TACAGTCCTCGTAGCTCTTGC
**Esrrg**	AAGATCGACACATTGATTCCAGC	CATGGTTGAACTGTAACTCCCAC
**aMHC**	GCCCAGTACCTCCGAAAGTC	GCCTTAACATACTCCTCCTTGTC
**bMHC**	ACTGTCAACACTAAGAGGGTCA	TTGGATGATTTGATCTTCCAGGG
**Mdm2**	TGTCTGTGTCTACCGAGGGTG	TCCAACGGACTTTAACAACTTCA
**Mfn1**	ATGGCAGAAACGGTATCTCCA	CTCGGATGCTATTCGATCAAGTT
**Mfn2**	ACCCCGTTACCACAGAAGAAC	AAAGCCACTTTCATGTGCCTC
**Myl2**	AGGACGAGTGAACGTGAAAAAT	GATTGCCGGTAACGTCAGGG
**Myl3**	TGGGGAAGCCAAAACAGGAAG	AGCCATCAGTTTCTCTACCTCA
**Mylk3**	GTGTGCCAAGGCATGGATCA	CCTCCTGTAGTCAGTACATGGT
**Myc**	ATGCCCCTCAACGTGAACTTC	CGCAACATAGGATGGAGAGCA
**Ndufa5**	ATGGCGGGCTTGCTGAAAA	GCTGCATGTTTAGGAAAGTGCTT
**Ndufa6**	TCGGTGAAGCCCATTTTCAGT	CTCGGACTTTATCCCGTCCTT
**Ndufa8**	GGAGCTGCCAACTCTGGAAG	CCAGCGGCACAGCATAAAC
**Npm1**	ATGGAAGACTCGATGGATATGGA	ACCGTTCTTAATGACAACTGGTG
**Opa1**	CGACTTTGCCGAGGATAGCTT	CGTTGTGAACACACTGCTCTTG
**p53**	CCCCTGTCATCTTTTGTCCCT	AGCTGGCAGAATAGCTTATTGAG
**Pgc-1a**	TATGGAGTGACATAGAGTGTGCT	CCACTTCAATCCACCCAGAAAG
**Pink1**	TTCTTCCGCCAGTCGGTAG	CTGCTTCTCCTCGATCAGCC
**Pln**	AAAGTGCAATACCTCACTCGC	GGCATTTCAATAGTGGAGGCTC
**Ppara**	AGAGCCCCATCTGTCCTCTC	ACTGGTAGTCTGCAAAACCAAA
**Ryr2**	ATGGCTTTAAGGCACAGCG	CAGAGCCCGAATCATCCAGC
**Sdha**	GGAACACTCCAAAAACAGACCT	CCACCACTGGGTATTGAGTAGAA
**Sdhb**	AATTTGCCATTTACCGATGGGA	AGCATCCAACACCATAGGTCC
**Tcap**	GATGCGCCTGGGTATCCTC	GATCGAGACAGGGTACGGC
**Tnnc1**	GCGGTAGAACAGTTGACAGAG	CCAGCTCCTTGGTGCTGAT
**Tnni3**	TCTGCCAACTACCGAGCCTAT	CTCTTCTGCCTCTCGTTCCAT
**Tnnt2**	CAGAGGAGGCCAACGTAGAAG	CTCCATCGGGGATCTTGGGT
**Tpm2**	AAGTCGCTGATAGCCTCAGAG	GGTCTGGTGTATCTCCACGTTC
**Tpm3**	GTCCCGTTGCCGAGAGATG	CTCGTCGCTAATGGCCTTGTA
**Tpm4**	CCGGAGTAGTGGGGCAAATAC	GGCGCGAAGAAGCAAAAGC
**Ttn**	GACACCACAAGGTGCAAAGTC	CCCACTGTTCTTGACCGTATCT
**Uqcr11**	TGCTGCGCAGGTTTCTAGGC	TCCACAGGACTACGCTGTGT

### Human cardiac samples

Human studies were conducted with the approval of the University Health Network Research Ethics Board (protocol 10–0703-TE). Informed written consent was obtained from patients with end-stage heart failure before the insertion of a left ventricular assist device. We purchased certified total RNA from normal adult human hearts from BioChain (Newark, CA 94560, USA).

## Results

### Acute genetic ablation of Mdm2 increases p53 protein stability

**Fig 1:**
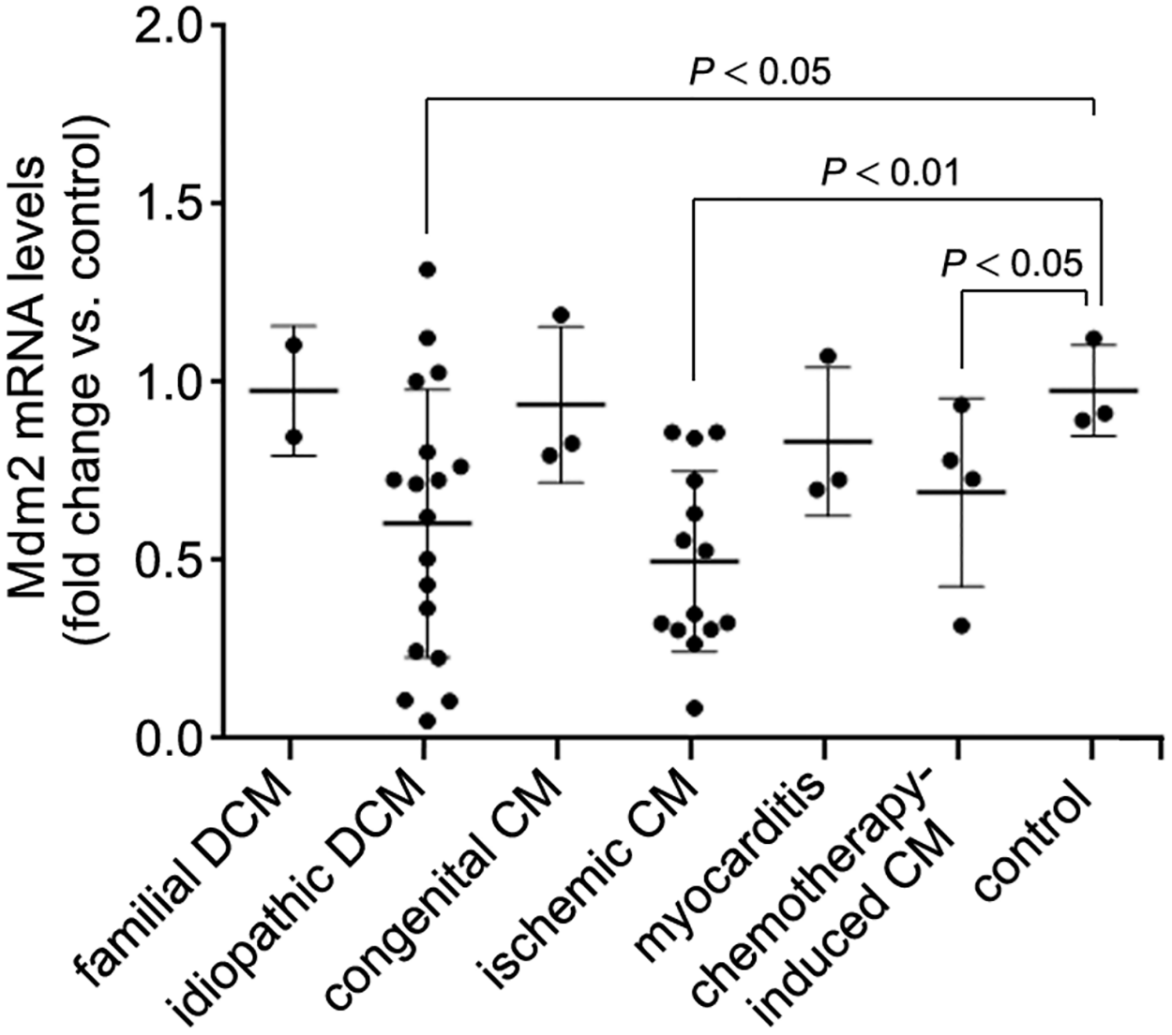
Down-regulation of Mdm2 mRNA levels in human end-stage heart failure determined by RT-qPCR. DCM, dilated cardiomyopathy. CM, cardiomyopathy. Control, normal human heart. Data are means±s.e.m.

**Fig 2:**
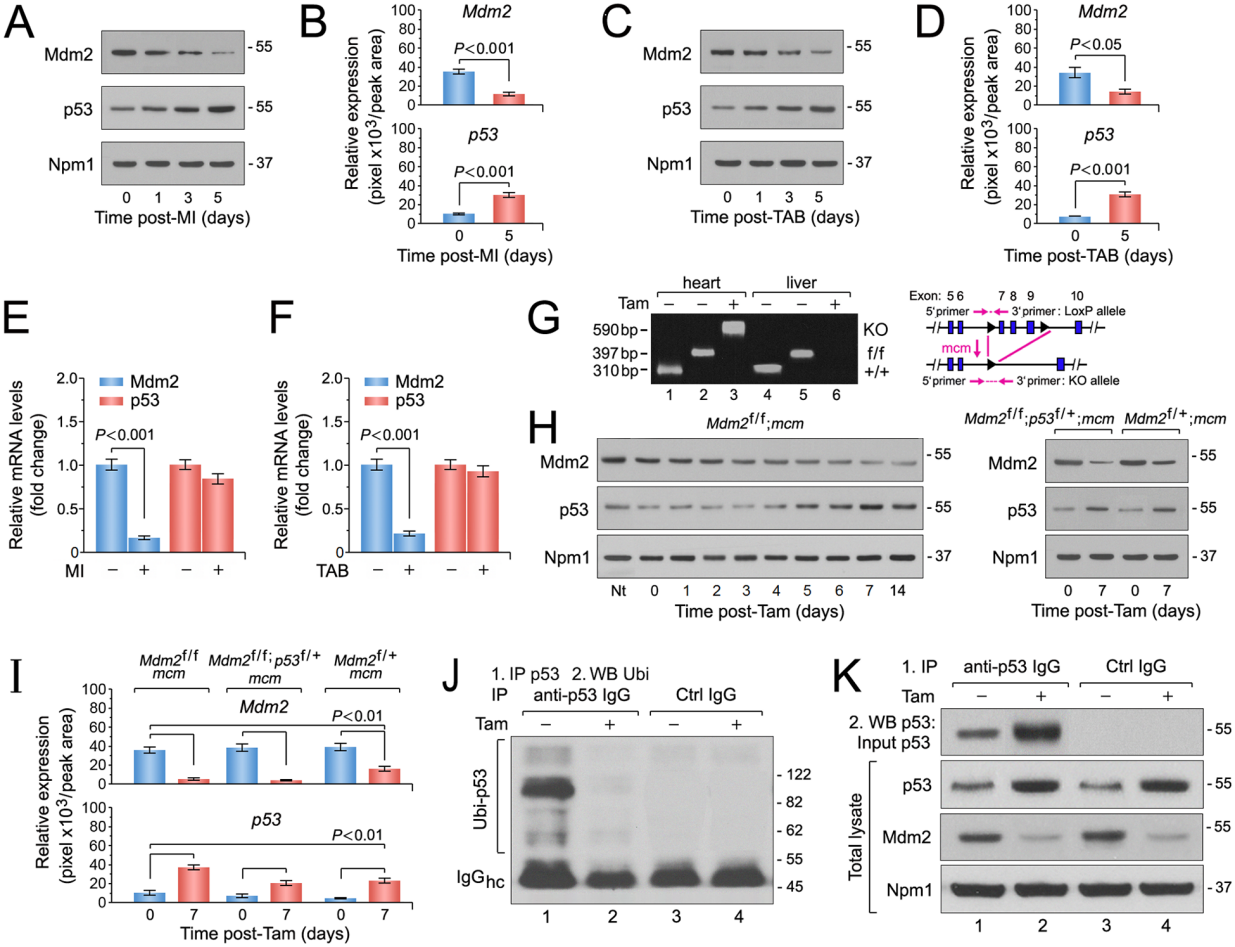
The E3 ubiquitin ligase Mdm2 is indispensable for the negative regulation of p53 protein stability in adult cardiomyocytes *in vivo*. Ctrl, control. Hc, heavy chains. IgG, immunoglobulin G. IP, immunoprecipitation. Tam, 4-hydroxytamoxifen. Ubi, ubiquitin. WB, Western blot. Numbers on the right indicate the relative molecular protein weight in kilodalton. **(A)** Mdm2 and p53 protein levels after ischemic injury (myocardial infarction, MI). Immunoblot analysis of left ventricular extracts (60 ug total protein/lane) of C57BL/6J wild type mice at the indicated time points was performed employing anti-Mdm2 and anti-p53 antibodies as indicated on the left. Animals were 13 weeks old at the time of analysis. For normalization, Western blots were probed with anti-nucleophosmin (Npm1). One representative immunoblot of 3 independent experiments is shown. **(B)** Protein levels shown in Fig 2A were quantified with ImageJ software. *n* = 3. **(C)** Mdm2 and p53 protein levels after acute pressure overload (TAB). Immunoblot analysis of left ventricular extracts (60 ug total protein/lane) of C57BL/6J wild-type mice at the indicated time points was performed employing anti-Mdm2 and anti-p53 antibodies as indicated on the left. Animals were 13 weeks old at the time of analysis. One representative immunoblot of 3 independent experiments is shown. **(D)** Quantification of protein levels shown in Fig 2C. *n* = 3. **(E)** Transcript levels of endogenous Mdm2 and p53 in 10-week-old wild-type mice post-MI as analyzed by RT-qPCR. *n* = 4. **(F)** RT-qPCR analysis of endogenous Mdm2 and p53 transcript levels in 10-week-old wild-type mice post-TAB as analyzed by RT-qPCR. *n* = 4. **(G)** Heart-specific deletion of *Mdm2*. Schematic structure of the floxed alleles of *Mdm2* (right panel). Genomic PCR results (left panel) of DNA isolated from LV tissue or liver control samples of wild-type (wt), vehicle injected control *Mdm2*^f/f^;*mcm* (-Tam) and *Mdm2*^f/f^;*mcm* mice at 7 days post-Tam (+Tam). Animals were 12 weeks old at the time of analysis. Numbers on the left refer to amplicon sizes in base pairs (bp). lane 1: *Mdm2*^+/+^;*mcm*, LV, -Tam. lane 2: *Mdm2*^f/f^;*mcm*, LV, -Tam. Lane 3: *Mdm2*^f/f^;*mcm*, LV +Tam. Lane 4: *Mdm2*^+/+^;*mcm*, liver, -Tam. lane 5: *Mdm2*^f/f^;*mcm*, liver, -Tam. Lane 6: *Mdm2*^f/f^;*mcm*, liver, +Tam. One representative result of 3 independent experiments is shown. **(H)** Immunoblot analysis of Mdm2 and p53 levels in left ventricular extracts (60 ug total protein/lane) of *Mdm2*^f/f^;*mcm* (left panel), *Mdm2*^f/+^;*mcm* and *Mdm2*^f/f^;*p53*^f/+^;*mcm* mice (right panel) employing specific antibodies as indicated on the left. Animals were 13 weeks old at the time of analysis. One representative immunoblot of 3 independent experiments is shown. **(I)** Quantification of protein levels of endogenous Mdm2 and p53 in the indicated strains shown in Fig 2H. *n* = 3. **(J and K)** Mdm2 regulates p53 protein stability in the adult mouse heart by regulation of its ubiquitin-mediated proteasomal degradation. **(J)** At 7d post-Tam, *Mdm2*^f/f^;*mcm* mice were intraperitoneally injected with the proteasomal inhibitor MG132 (30 mmol/kg body weight) for 6 hours. Left ventricular lysates were immunoprecipitated (IP) with anti-p53 antibodies or normal rabbit IgG. Ubiquitinated p53 proteins in the immunoprecipitates were identified by immunoblotting with antibodies to ubiquitin. One representative immunoblot of 3 independent experiments is shown. IgG, immunoglobulin G. IP, immunoprecipitation. Ubi, ubiquitin. WB, Western blot. **(K)** Levels of endogenous Mdm2 and p53 proteins in total left ventricular extracts prepared from *Mdm2*^f/f^;*mcm* mice in the presence and absence of Tam (middle and bottom panels). Samples were subjected to anti-p53 immunoprecipitations and Western blots were probed with anti-p53 antibodies (top panel). The same samples as in Fig 3J were analyzed. The mice were 12 weeks old at the end of the experiment. One representative immunoblot of 3 independent experiments is shown. Fig 2 data are means±s.e.m.

We analyzed mRNA isolated from LV samples of patients with end-stage HF of varying etiologies. When compared to mRNA isolated from healthy hearts, Mdm2 gene expression was significantly decreased in samples from patients with idiopathic dilated cardiomyopathy (DCM), ischemic cardiomyopathy and chemotherapy-induced cardiomyopathy (*P* < 0.05) (Fig 1), suggesting a correlation between Mdm2 expression and end-stage HF of these etiologies. Hence, to elucidate the potential function of the p53/Mdm2 circuitry in cardiomyocytes, we examined their protein expression and mRNA levels in left ventricular samples derived from C57BL/6J wild-type mice subjected to myocardial infarction (MI) or trans-aortic banding (TAB). We observed that Mdm2 protein levels were significantly decreased after MI (*P* < 0.001) (Fig 2A and 2B) and markedly downregulated after TAB in wild-type mice (*P* < 0.05) (Fig 2C and 2D). In contrast, p53 protein contents were significantly increased in both heart failure models (Fig 2A–2D). However, the same was not observed in p53 mRNA levels post-MI (Fig 2E) and post-TAB (Fig 2F) (*P* > 0.05) demonstrating that p53 protein expression is regulated post-transcriptionally. These data illustrate that when chronically exposed to ischemic or mechanical stress, the murine heart undergoes maladaptive changes including impaired Mdm2 expression that is inversely correlated to p53 protein levels. These findings are compatible with the view that Mdm2 exerts an activity in adult cardiomyocytes that maintains p53 at low levels and inhibits its activation. However, it can be robustly activated in response to ischemic and biomechanical stress, contributing to ventricular remodeling and the development of heart failure [52–57].

To explore the reciprocal regulation of p53 by Mdm2 mechanistically *in vivo*, we crossed transgenic mice expressing Cre recombinase flanked by mutated estrogen receptors (MerCreMer; *mcm*) with mice carrying loxP flanked alleles (f/f) of *Mdm2* [58] to obtain *Mdm2*^f/f^;*mcm* animals. The day of the last injection was arbitrarily set to 0. Four consecutive daily intraperitoneal Tam injections induced genetic ablation of Mdm2 at 7 days post-Tam (Fig 2G). We observed a marked increase in p53 protein levels with an overlapping decrease in Mdm2 expression in left ventricular extracts from *Mdm2*^f/f^;*mcm* mice at 7 days post-Tam (Fig 2H) indicating a high recombination efficiency (> 90%) as determined by densitometric immunoblot analysis (Fig 2I). As expected, we detected approximately 50% less p53 protein in LV extracts of Tam-treated *Mdm2*^f/+^;*mcm* and *Mdm2*^f/f^;*p53*^f/+^;*mcm* mice (Fig 2H and 2I). Based on these findings, we surmised that Mdm2 regulates p53 protein stability in differentiated cardiomyocytes. To investigate this further, endogenous p53 was immunoprecipitated with anti-p53 antibodies from left ventricular lysates of *Mdm2*^f/f^;*mcm* that were intraperitoneally injected with the proteasome inhibitor MG132 (Fig 2J) [49,50]. Western blots were subsequently reacted with antibodies specifically recognizing ubiquitin (Fig 2K). The immunoblot analysis clearly demonstrates the presence of ubiquitinated p53 in vehicle-injected *Mdm2*^f/f^;*mcm* animals that is absent in Mdm2-deficient hearts. These findings imply that Mdm2 is a major E3-ubiquitinase for p53 in the adult heart at baseline.

### Acute cardiac-specific ablation of Mdm2 is associated with pathological cardiac hypertrophy and early death

**Fig 3:**
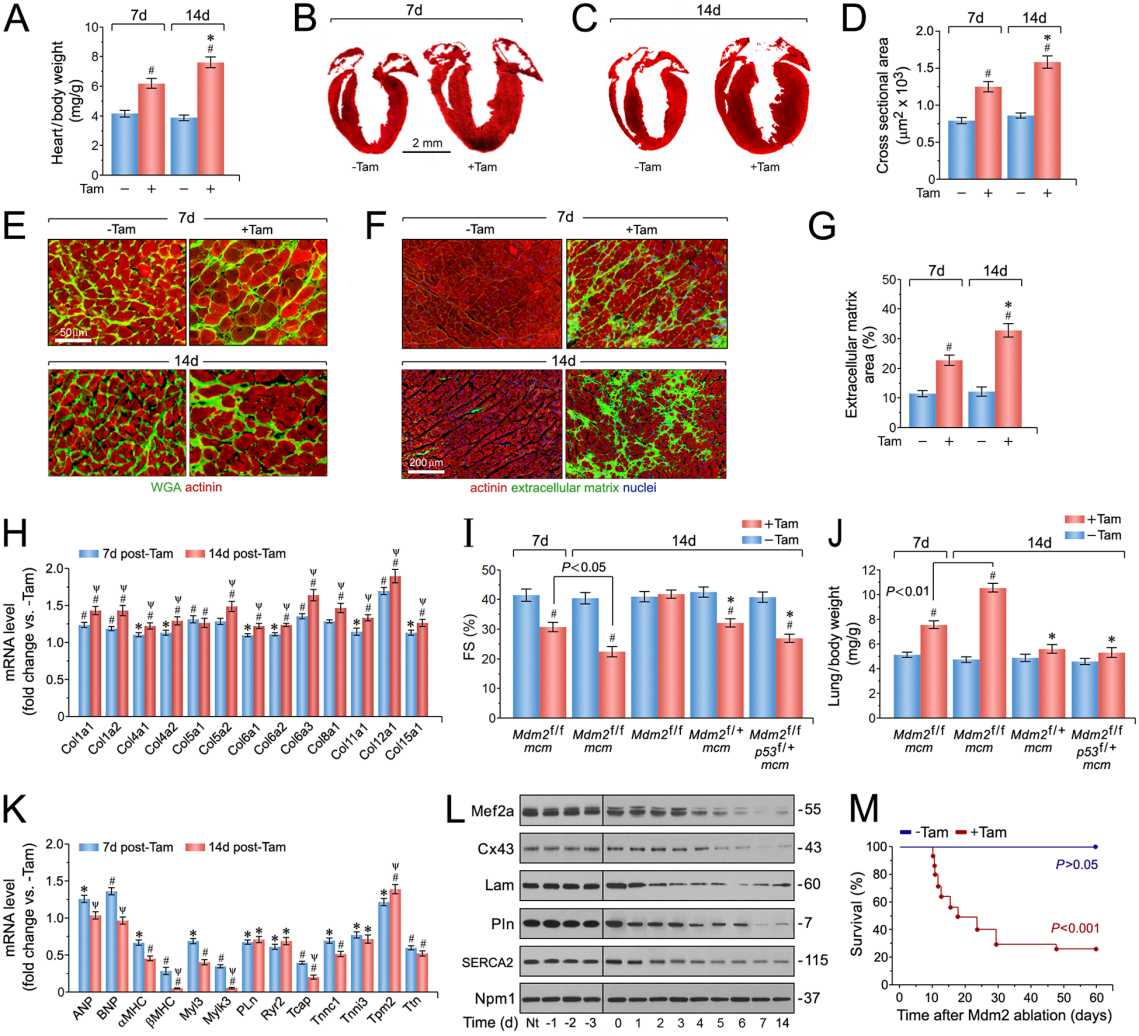
Ablation of Mdm2 is associated with the development of concentric hypertrophy and cardiac dysfunction. (A) Heart-weight corrected for body weight in *Mdm2*^f/f^;*mcm* mice at 7d and 14 days post-Tam. *n* = 24. ^#^*P* < 0.01 vs. -Tam. *P < 0.01 vs. *Mdm2*^f/f^;*mcm* at 7 days post-Tam. (B) Masson staining of longitudinal cardiac sections of *Mdm2*^f/f^;*mcm* mice at 7 days post-Tam. (C) Masson staining of longitudinal cardiac sections of *Mdm2*^f/f^;*mcm* mice at 14 days post-Tam. (D) Quantification of cross-sectional area of adult cardiomyocytes in *Mdm2*^f/f^;*mcm* mice shown in Fig 2E. *n* = 6–8. ^#^*P* < 0.01 vs. -Tam. *P < 0.05 vs. *Mdm2*^f/f^;*mcm* at 7 days post-Tam. (E) Immunofluorescence microscopy of wheat germ agglutinin (WGA, green; top panel) and cardiomyocyte-specific anti-actinin (red; bottom panel) stained left ventricular sections from *Mdm2*^f/f^;*mcm* mice. (F) Immunofluorescence microscopy employing WGA staining (green) of the extracellular matrix, cardiomyocyte-specific anti-actinin (red), and Dapi (blue) to visualize nuclear DNA in left ventricular sections from *Mdm2*^f/f^;*mcm* mice. (G) Quantification of extracellular matrix area indicative of left ventricular fibrosis in *Mdm2*^f/f^;*mcm* mice shown in Fig 2F. *n* = 4. ^#^*P* < 0.01 vs. -Tam. *P < 0.05 vs. *Mdm2*^f/f^;*mcm* at 7 days post-Tam. (H) Transcript levels of differentially expressed collagen types in Tam-treated *Mdm2*^f/f^;*mcm* mice as determined by RT-qPCR at 7 days and 14 days post-Tam. *n* = 4. ^#^*P* < 0.01 vs. -Tam. *P < 0.05 vs. -Tam. ^Ψ^*P* < 0.05 vs. 7d +Tam. (I) Fractional shortening (FS) determined by M-mode echocardiography of the indicated strains at 7 and 14 days post-treatment with Tam or vehicle. *n* = 6. ^#^*P* < 0.01 vs. -Tam. *P < 0.05 vs. *Mdm2*^f/f^;*mcm* at 14 days post-Tam. (J) Lung/body weight ratios in various Mdm2/p53 mutant mice at 7 and 14 days after Tam treatment. *n* = 6. ^#^*P* < 0.01 vs. -Tam. *P < 0.05 vs. *Mdm2*^f/f^;*mcm* at 14 days post-Tam. (K) Levels of hypertrophic marker genes in *Mdm2*^f/f^;*mcm* mice as analyzed by qRT-PCR at 7 days and 14 days post-Tam. *n* = 4. ^#^*P* < 0.01 vs. -Tam. *P < 0.05 vs. -Tam. ^Ψ^*P* < 0.05 vs. 7d +Tam. (L) Immunoblot analysis of cardiac-specific gene expression in left ventricular samples from *Mdm2*^f/f^;*mcm* mice. Nt, no treatment. Western blots were repeated at least once with similar results. (M) Acute genetic ablation of Mdm2 evokes premature death. Kaplan-Meier survival curves of conditional *Mdm2*^f/f^;*mcm* mice. *n* = 20. Fig 3A to Fig 3M: 12-week-old mice (7 days post-Tam) and 13-week-old mice (14 days post-Tam) were analyzed. Fig 3 data are means±s.e.m.

To determine the physiological consequence of Mdm2 ablation, cardiac morphology, and function in *Mdm2*^f/f^;*mcm* mice were assessed in the presence and absence of Tam. We found that *Mdm2*^f/f^;*mcm* developed normally without an obvious cardiac phenotype in the absence of Tam (Fig 3A to 3C). In contrast, *Mdm2*^f/f^;*mcm* mice developed concentric cardiac hypertrophy as early as 7 days post-Tam, with significant wall thickening (Fig 3A and 3B), and an average increase of 98% (*P* < 0.001) (Fig 3A) in heart weight/body weight (HBW) ratios at 14 days post-Tam in comparison to vehicle controls (Fig 3A and 3C). To determine whether the increased HBW ratio in *Mdm2*^f/f^;*mcm* hearts was due to an increase in cell size, the width of cardiomyocytes was measured in wheat germ agglutinin (WGA)-stained specimen of the left ventricular free wall in these animals. In these samples, the cross-sectional area of Mdm2-deficient cardiomyocytes was already significantly elevated after 7 days (P < 0.01) (Fig 3D and 3E). At 14 days after Tam administration, there was a 1.9-fold increase in cardiomyocyte width in *Mdm2*^f/f^;*mcm* mice (*P* < 0.01) compared with vehicle injected controls (Fig 3D and 3E). *Mdm2*^f/f^;*mcm* hearts also exhibited extensive interstitial fibrosis over controls at 7 days and 14 days post-Tam (2.7-fold higher) (*P* < 0.01) (Fig 3F and 3G). We then measured mRNA levels of different collagens in left ventricular samples and found the transcript levels of several collagen (Col) classes including Col type I, Col IV and Col VI, were significantly increased in *Mdm2*^f/f^;*mcm* at 7 days and 14 days post-Tam when compared to vehicle-treated controls (*P* < 0.01) (Fig 3H). Examination of cardiac function by echocardiography on viable adult *Mdm2*^f/f^;*mcm* mice post-Tam revealed significant decreases in fractional shortening as early as 7 days post-Tam, that deteriorated further by 14 days (23 + 2.4%), in comparison to vehicle-injected *Mdm2*^f/f^;*mcm* (41 + 4.3%; *P* < 0.01) and Tam-treated *Mdm2*^f/f^ control groups (Fig 3I). Importantly, FS was significantly higher in hearts of Tam-treated *Mdm2*^f/+^;*mcm and Mdm2*^f/f^;*p53*^f/+^;*mcm* mice when compared to *Mdm2*^f/f^;*mcm* animals post-Tam (*P* < 0.05) (Fig 3I). Moreover, lung/body weight ratios were significantly increased in *Mdm2*^f/f^;*mcm* mice at 7 days and 14 days post-Tam (*P* < 0.01) (Fig 3J). This was not observed in the *Mdm2*^f/+^;*mcm and Mdm2*^f/f^;*p53*^f/+^;*mcm* animals (*P* > 0.05). Thus, *Mdm2*^f/f^;*mcm* mice developed congestive heart failure after Tam administration, that appeared to be due to a higher p53 activity.

To explore the molecular basis for the cardiac abnormalities in *Mdm2*^f/f^;*mcm* mice exposed to Tam, RT-qPCR was carried out on total RNA isolated from left ventricular tissue samples at 7 days and 14 days post-Tam. Notably, we detected altered transcript levels of structural and stress-responsive cardiac genes as early as 7 days post-Tam. In particular, significant decreases in expression of genes regulating Ca^2+^-handling (*Pln*, *Ryr2*), and contractile proteins (for example, *Mylk3*, *Tnnc1*, *Tnni3*, *Ttn*) were observed and in keeping with the cardiac dysfunction of Mdm2-deficient hearts (*P* < 0.01) (Fig 3K). Intriguingly, transcripts for atrial natriuretic factor (ANF), and brain natriuretic peptide (BNP), canonical markers of cardiac hypertrophy and heart failure, were initially increased by 7 days, but dropped to control levels at 14 days (*P* < 0.05) in *Mdm2*^f/f^;*mcm* mice after Tam-administration, whereas alpha and beta myosin heavy chain (a/bMHC) mRNA were significantly downregulated by -39% and -47% (*P* < 0.001) (Fig 3K). All these effects were further corroborated by immunoblot analysis of selected cardiac genes in *Mdm2*^f/f^;*mcm* mice over a time course of 14 days in the presence and absence of Tam (Fig 3L). Importantly, 50% of *Mdm2*^f/f^;*mcm* mice died unexpectedly by 18 days post-Tam, and more than 75% of animals succumbed within 30 days that was not observed in vehicle-treated *Mdm2*^f/f^;*mcm* controls (*P* < 0.001) (Fig 3M). In contrast, heterozygosity of p53 was sufficient to rescue the lethality of *Mdm2*^f/f^;*mcm* mice exposed to Tam in the same observation period (Fig 3M). Early death of *Mdm2*^f/f^;*mcm* mice could also be rescued by heterozygosity of Mdm2, demonstrating a specific gene dose effect for Mdm2 in the adult heart.

### Genome-wide expression profiling of Mdm2-regulated transcripts

**Fig 4:**
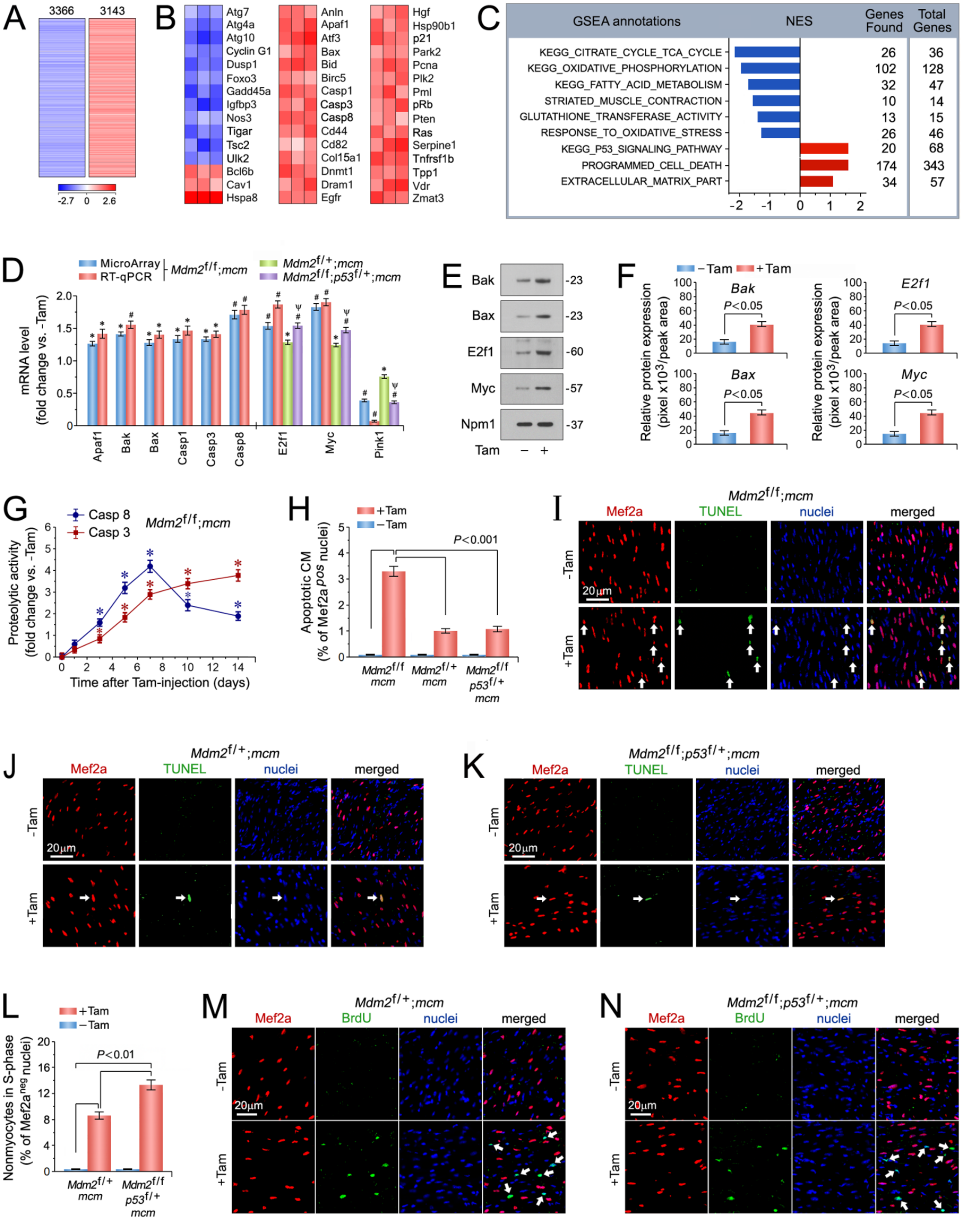
p53 is spontaneously active in Mdm2-deficient cardiomyocytes and induces apoptosis. (A) Genome-wide messenger RNA (mRNA) microarray profiling reveals that conditional genetic ablation of Mdm2 induces profound alterations in the cardiac transcriptome indicating a high degree of complexity associated with Mdm2-deficiency. Heat map of unsupervised hierarchical cluster analysis at a high confidence threshold identifies 6,509 transcripts (rows) out of 26,166 individual genes (columns) that were enriched in the hearts of *Mdm2*^f/f^;*mcm* mice post-Tam relative to vehicle-injected controls. Values (log_2_ expression) are shown by color and intensity of shading. Blue, repressed. Red, induced. *n* = 3 biological replicates. *P* < 0.01. Fold change >1.3. Mice (13 weeks old) were analyzed at 14 days post-Tam. (B) Heat maps examining the impact of genomic modifications in Mdm2-deficient hearts (columns) on previously validated p53 target genes (rows). Values, log_2_ expression. *n* = 3. *P* < 0.01. Fold change >1.3. (C) Gene Set Enrichment Analysis (GSEA) of biological processes among all differentially expressed transcripts, assessed by overrepresentation of GSEA terms for the biological function of each transcript in hearts of *Mdm2*^f/f^;*mcm* mice post-Tam. NES, normalized enrichment score. NES have False Discovery Rate (FDR) *q*-values <0.1. Blue, inhibited processes. Red, activated processes. (D) Expression levels of key genes selected from top-ranked gene sets identified in Fig 4A, involved in the regulation of cardiomyocyte apoptosis in the indicated Mdm2 and p53 mutant mice as analyzed by RT-qPCR at 7 days post-Tam. *n* = 3. *P < 0.05 vs. -Tam. ^#^*P* < 0.01 vs. -Tam. Ψ < 0.05 vs. *Mdm2*^f/+^;*mcm* and *Mdm2*^f/f^;*mcm*. (E) Immunoblot analysis of master regulators of apoptosis in *Mdm2*^f/f^;*mcm* mice at 7 days post-Tam employing specific antibodies as indicated on the left. One representative immunoblot of 3 independent experiments is shown. (F) Protein levels shown in Fig 4E were quantified with ImageJ software. *n* = 3. (G) Time course of caspase 3/8 activation in *Mdm2*^f/f^;*mcm* mice. *n* = 4. *P < 0.05 vs. -Tam. (H) Quantification of apoptotic cardiomyocytes shown in Fig 4I-K. *n* = 4. (I to K) Analysis of apoptosis in left ventricular cardiomyocytes (white arrows) in various Mdm2 and p53 mutant strains at 7 days post-Tam by immunofluorescence microscopy and terminal deoxynucleotidyl transferase dUTP nick end labeling (TUNEL) assay. Red, anti-Mef2a. Green, TUNEL. Blue, DAPI stain of nuclear genomic DNA. (L) Induction of cell cycle entry and DNA synthesis is restricted to non-myocytes in the indicated Mdm2 and p53 mutant strains. Quantification of non-myocytes in S phase of the cell cycle shown in Fig 4M and N. *n* = 4. (M, N) Analysis of DNA replication in left ventricular non-myocytes (white arrows) in the indicated Mdm2 and p53 mutant strains at 7 days post-Tam by immunofluorescence microscopy. Red, anti-Mef2a. Green, BrdU. Blue, DAPI stain of nuclear genomic DNA. Mice were 13 weeks old at 14 days post-Tam (Fig 4A to C). Animals were 12 weeks old mice at 7 days post-Tam (Fig 4C to N). **Abbreviations of gene names in** Fig 4B: **Anln**, anillin actin binding protein; **Apaf1**, apoptotic peptidase activating factor 1; **Atf3**, activating transcription factor 3; **Atg7**, autophagy related 7; **Atg4a**, autophagy related 4A cysteine peptidase; **Atg10**, autophagy related 10; **Bax**, BCL2-associated X protein; **Bcl6b**, B cell CLL/lymphoma 6, member B; **Bid**, BH3 interacting domain death agonist; **Birc5**, baculoviral IAP repeat-containing 5; **Casp1**, caspase 1; **Cav1**, caveolae protein caveolin 1; **Cd44**, CD44 antigen; **Cd82**, CD82 antigen; **Col18a1**, collagen type XVIII alpha 1; **Cyclin G1** (Ccng1); **Dnmt1**, DNA methyltransferase (cytosine-5) 1; **Dram1**, DNA-damage regulated autophagy modulator 1; **Dusp1**, dual specificity phosphatase 1; **Egfr**, epidermal growth factor receptor; **Foxo3**, forkhead box O3; **Gadd45a**, growth arrest and DNA-damage-inducible 45 alpha; **Hgf**, hepatocyte growth factor; **Hspa8**, heat shock protein 8; **Hsp90b1**, heat shock protein 90 beta (Grp94) member 1; **Igfbp3**, insulin-like growth factor binding protein 3; **Nos3**, endothelial cell nitric oxide synthase 3; **p21**, cyclin-dependent kinase inhibitor 1A (*Cdkn1a*); **Park2** (parkin), Parkinson disease autosomal recessive, juvenile 2; **Pcna**, proliferating cell nuclear antigen; **Plk2**, polo-like kinase 2; **Pml**, promyelocytic leukemia; **pRb**, retinoblastoma 1 (*Rb1*); **Pten**, PTEN induced putative kinase 1; **Ras**, Harvey rat sarcoma virus oncogene (*Hras*); **Serpine1**, serine (or cysteine) peptidase inhibitor clade E member 1; **Tigar**, Trp53 induced glycolysis regulatory phosphatase; **Tnfrs1b**, TNF receptor superfamily member 1B; **Tpp1**, tripeptidyl peptidase I; **Tsc2**, tuberous sclerosis 2; **Ulk2**, unc-51 like kinase 2; **Vdr**, vitamin D receptor; **Zmat3**, zinc finger matrin-type 3. Fig 4 data are means±s.e.m.

To uncover mechanisms responsible for the cardiac phenotype induced by Mdm2-deficiency at the transcriptome level, genome-wide RNA expression profiling was performed on left ventricular samples from *Mdm2*^f/f^;*mcm* mice in the presence and absence of Tam. Acute ablation of Mdm2 induced a broad derangement of the cardiac transcriptome (Fig 4A). At 14 days after Tam administration, 6,509 of 26,166 individual genes were differentially regulated in the hearts of *Mdm2*^f/f^;*mcm* mice (false discovery rate < 0.01%) in comparison to vehicle-injected *Mdm2*^f/f^;*mcm* mice. Moreover, significant enrichment of canonical p53 target genes [59] in Mdm2-deficient hearts indicates an association between activated p53 and Mdm2 ablation (*P* < 0.01) (Fig 4B).

To identify biological processes underlying the development of the cardiac phenotype in the absence of Mdm2 at 14 days post-Tam, Gene set enrichment analysis (GSEA) analysis was performed. GSEA identified several highly significantly gene clusters with similar biological functions in the mutant Mdm2 transcriptome that were known to be critically involved in the regulation of normal heart function (*P* < 0.01) (Fig 4C). The top ranked GSEA terms (Fig 4C) with negative (downregulated genes, blue) normalized enrichment scores (NES) were the p53 signaling pathway, tricarboxylic acid cycle (TCA), oxidative phosphorylation, fatty acid metabolism, striated muscle contraction (as analyzed in Fig 3J and 3K), glutathione transferase, and response to oxidative stress. Programmed cell death and extracellular matrix (as analyzed in Fig 3F, 3G and 3H) were highly enriched gene sets containing upregulated transcripts (positive NES, red) (Fig 4C). The development of a cardiac phenotype occurred as early as 7 days in these animals (Fig 3A–3K). Therefore, we decided to test the physiological relevancy of main regulators in these gene sets for their ability to drive the development of heart failure associated with Mdm2 loss at an earlier time point at 7 days post-Tam.

### Spontaneous activation of p53 in Mdm2-deficient cardiomyocytes induces apoptosis

With the observation of elevated p53 protein levels in hearts of *Mdm2*^f/f^;*mcm* mice exposed to Tam (Fig 2H), we initially investigated the sensitivity of this strain to p53-triggered oxidative stress and apoptosis. Top ranked genes included direct proapoptotic p53 target genes Apaf1 [60–62], caspases 1 [63], 3 [64,65] and 8 [66–68], E2f1 [69–72], and c-Myc (Myc) [71,73] as well as Bax and Bak, key p53-interacting factors during transcription-independent p53 cell death [74,75]. E2f1 [72] and Myc [73] are ubiquitously expressed, highly pleiotropic transcription factors. In mitotic cells, they promote proliferation, apoptosis, DNA damage, sensitivity to reactive oxygen species (ROS), and the oxidative stress response [70,71,73]. Significant upregulation (*P* < 0.05) of all of these factors in Tam-injected *Mdm2*^f/f^;*mcm* was confirmed by real time PCR (RT-qPCR) (Fig 4D) and immunoblot analysis (Fig 4E and 4F) at 7 days post-Tam.

Next, we analyzed whether mRNA expression of E2f1, Myc and Pink1 are regulated by p53 or by Mdm2 independently of p53. Thus, RT-qPCR was carried out on total RNA isolated from Tam-treated *Mdm2*^f/+^;*mcm* and *Mdm2*^f/f^;*p53*^f/+^;*mcm* mice. We observed, that transcript levels of E2f1 and Myc were significantly higher in hearts of *Mdm2*^f/f^;*p53*^f/+^;*mcm* mice as compared to *Mdm2*^f/+^;*mcm* animals post-Tam (*P* < 0.05) (Fig 4D). Moreover, Pink1 mRNA expression was lower in LV samples from *Mdm2*^f/f^;*p53*^f/+^;*mcm* mice in comparison to *Mdm2*^f/+^;*mcm* animals (*P* < 0.05) (Fig 4D). These findings are consistent with the hypothesis that gene expression of E2f1, Myc and Pink1 regulated by p53, and occurs in a Mdm2-independent manner.

Transcriptional activation of caspase-3 and 8 in a p53-dependent fashion has been well established in previous studies [65,67]. Thus, we examined the kinetics of caspase-8 (extrinsic cell death pathway) and caspase-3 (intrinsic apoptosis) [76] activation to determine their relationship to the development of heart failure in the absence of Mdm2. Spectrophotometric measurement of caspase-dependent proteolytic activities in left ventricular lysates were shown to be significantly induced in the presence of Tam within 3 days (caspase-8) and 5 days (caspase-3) (*P* < 0.05), and reached their maximum at 7 days (Fig 4G). Significant levels of caspase-3 and 8 activities were not detected in control mice (Fig 4G). Notably, we observed a marked increase in the number of TUNEL-positive cardiomyocyte nuclei in fixed left ventricular specimen from Tam-injected *Mdm2*^f/f^;*mcm* mice (3.3 ± 0.46%; *P* < 0.001) as compared to controls (0.1 ± 0.02%; *P* < 0.001) (Fig 4H and 4I). Importantly, reduction of p53 levels in Tam-treated *Mdm2*^f/+^;*mcm* (Fig 4H and 4J), and *Mdm2*^f/f^;*p53*^f/+^;*mcm* mice (Fig 4H and 4K) significantly reduced the degree of cardiomyocyte apoptosis in comparison to the *Mdm2*^f/f^;*mcm* strain (*P* < 0.001). We infer from this data, that p53 is a physiological relevant target of Mdm2. These findings suggest that induction of cardiomyocyte apoptosis by p53 due to loss of Mdm2, plays a role in the initiation phase of a process that culminates in left ventricular dysfunction and premature death.

We have previously reported, that homozygous genetic deletion of both p53 and Mdm2 in adult mice significant increases adult cardiomyocyte proliferation [77]. In contrast, CM in *Mdm2*^f/f^;*p53*^f/+^;*mcm* mice exhibited a markedly lower extent of cell cycle re-entry post-Tam. Notably, deficiency of p53 alone was insufficient to induce CM DNA replication [77]. To investigate whether heterozygosity for p53 in Mdm2-deficient mice induces DNA synthesis in adult CM, we intraperitoneally injected *Mdm2*^f/f^;*p53*^f/+^;*mcm* mice with BrdU, and analyzed fixed LV cryosections for evidence of CMs in S phase of the cell cycle at 7d post-Tam. *Mdm2*^f/+^;*mcm* mice were also included in this analysis, because CM cell cycle activity in this strain had not been analyzed in previous studies [77]. Immunofluorescence imaging repeatedly showed significant DNA synthesis of non-CM cell populations (Fig 4L). There was no evidence of CMs in S phase in *Mdm2*^f/f^;*p53*^f/+^;*mcm* and *Mdm2*^f/+^;*mcm* mice with or without Tam injection (Fig 4M and 4N). Thus, heterozygosity for p53 on an Mdm2 negative genetic background is insufficient to induce cell cycle re-entry.

### Mdm2-deficiency enhances oxidative stress in cardiomyocytes

**Fig 5:**
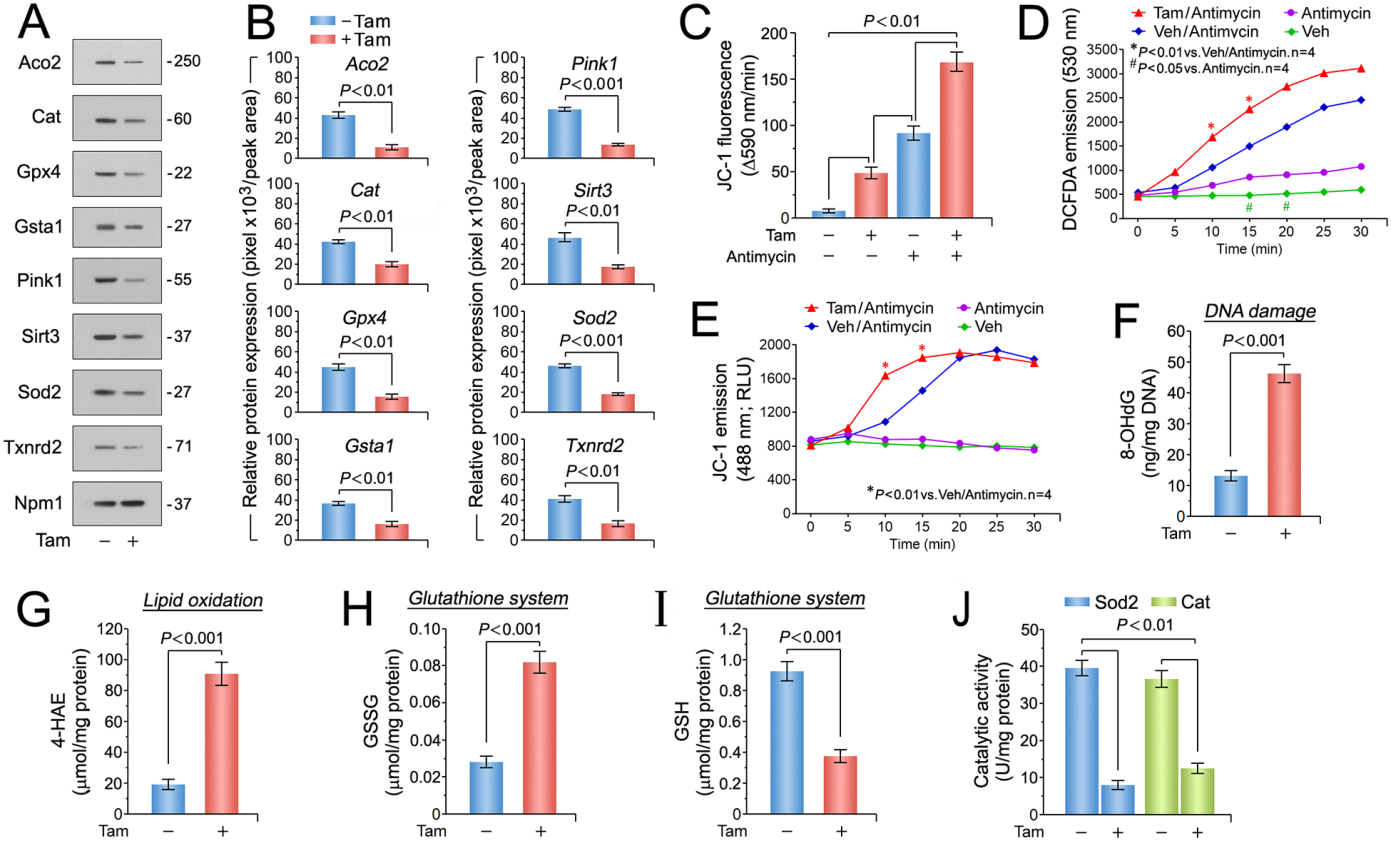
Enhanced oxidative stress and impaired antioxidant systems in cardiomyocytes lacking Mdm2. (A) Immunoblot analysis of key genes selected from top-ranked gene sets identified in Fig 3C involved in detoxification processes of reactive oxygen species (ROS). One representative immunoblot of 3 independent experiments is shown. Left ventricular samples from *Mdm2*^f/f^;*mcm* mice were analyzed at 7 days post-Tam. (B) Densitometric analysis of Western blot results shown in Fig 5A employing ImageJ software. Numbers at the bottom of each panel indicate fold-change. *n* = 3 (C) Isolated cardiac mitochondria from Tam-treated *Mdm2*^f/f^;*mcm* have a membrane potential (MMP) that is susceptible to ROS-induced depolarization. Mitochondria were mechanically isolated, incubated with JC-1 (5.0 ug/mL), and were then treated with antimycin (50 uM). JC-1 emission at 535/595 nm was recorded at 1 reading/min for 30 min using a fluorescence spectrophotometer. The rate between two time points (emission at 595 nm/min) was calculated in the most linear range of decline for JC-1 fluorescence intensity. *n* = 4. (D) DCFAD (dichlorofluorescin diacetate) emission data in isolated cardiac mitochondria from Tam-treated *Mdm2*^f/f^;*mcm* mice. *n* = 4. (E) JC-1 green emission data in isolated cardiac mitochondria from Tam-treated *Mdm2*^f/f^;*mcm* mice. RLU, relative light units. *n* = 4. (F) Higher oxidative genomic DNA damage in *Mdm2*^f/f^;*mcm* mice post-Tam when compared to vehicle-injected controls. Concentrations of 8-hydroxy-2’-deoxyguanosine (8-OHdG), a biomarker for oxidative DNA damage in the indicated strains was determined by a competitive enzyme-linked ELISA employing 8-OHdG antibodies. *n* = 4. (G) Significantly enhanced levels of 4-hydroxyalkenals (4-HAE), an indicator of ROS-dependent lipid peroxidation in *Mdm2*^f/f^;*mcm* mice post-Tam compared with control injected animals. *n* = 4. (H and I) Markedly reduced glutathione/oxidized glutathione (GSH/GSSG) ratios, an indicator of cardiac oxidative stress, in *Mdm2*^f/f^;*mcm* mice post-Tam compared to controls. *n* = 4. (J) Significantly downregulated mitochondrial superoxide dismutase (Sod2) and catalase (Cat) activities as determined spectrophotometrically in *Mdm2*^f/f^;*mcm* mice post-Tam. *n* = 4. Fig 5A to J: Left ventricular samples from 12-week-old mice were analyzed at 7 days post-Tam. Fig 5 data are means±s.e.m.

Enhanced oxidative stress associated with mitochondrial dysfunction has been well documented in patients with heart failure [78–80]. Since p53 [81], E2f1 [82], and Myc [83] play important roles in redox homeostasis and ROS generation, we analyzed the expression of the top-ranked antioxidant factors contained in the GSEA term “response to oxidative stress” (Fig 4*C*) by immunoblotting of left ventricular lysates. Pink1, mitochondrial *Aco2*, *Cat*, *Gpx4*, *Gsta1*, mitochondrial *Sirt3*, *Sod2*, and *Txnrd2* were markedly downregulated in *Mdm2*^*f/f*^;*mcm* post-Tam, but not in control mice (Fig 5A and 5B). Since mitochondrial complex I (CI) activity and mitochondrial membrane potential is impaired in the hearts of systemic Pink1 knockout mice [84,85], we evaluated the consequence of Mdm2 ablation on mitochondrial function. Production of reactive oxygen species (ROS) by the mitochondrial respiratory chain is a main contributor to the development of heart failure associated with oxidative stress [86]. Two production sites for ROS have been identified at mt C1 and mt C3 [87,88]. The production of ROS at these sites can be greatly enhanced by specific inhibitor, rotenone for C1 and antimycin for C3 [89]. Both compounds stimulate ROS generation by induction of a reverse-electron flux [90]. To analyze the integrity of the mitochondrial transmembrane potential (MMP), mitochondrial isolated from left ventricles derived from Tam-treated *Mdm2*^f/f^;*mcm* mice were labeled with the fluorochrome JC-1 (2.0 uM), and then exposed to antimycin (50 uM). As expected, we observed a ROS-dependent impairment of MMP in mitochondrial from Mdm2-deficient hearts compared relative to vehicle controls at baseline and in the presence of antimycin that indicates enhanced ROS production (*P* < 0.01) (Fig 5C to 5E). In agreement with these findings, we found 3.5-fold higher levels of oxidative stress indicators 8-hydroxy-2′-deoxyguanosine (8-OHdG) (Fig 5F), and 4.5-fold higher levels of 4-hydroxyalkenals (4-HAE) (*P* < 0.001) (Fig 5G) in Tam-treated *Mdm2*^f/f^;*mcm*. This data suggest that Mdm2 participates in the negative regulation of Pink1 protein expression to contribute to oxidative stress. GSH (-59%)/GSSG (+283%) ratios, key components in the cellular anti-oxidation, were also significantly decreased in Tam injected *Mdm2*^f/f^;*mcm* hearts over controls (*P* < 0.001) (Fig 5H and 5I). Finally, administration of Tam to *Mdm2*^f/f^;*mcm* mice markedly downregulated activities of ROS detoxifying enzymes Sod2 (-80%) and Cat (-65%; *P* < 0.01) (Fig 5J). Taken together, our results demonstrate that Mdm2-deficiency is accompanied by elevated oxidative stress within the mitochondria.

### Mdm2 ablation inhibits the Pgc-1/Ppar/Esrr axis and leads to broad mitochondrial dysfunction

**Fig 6:**
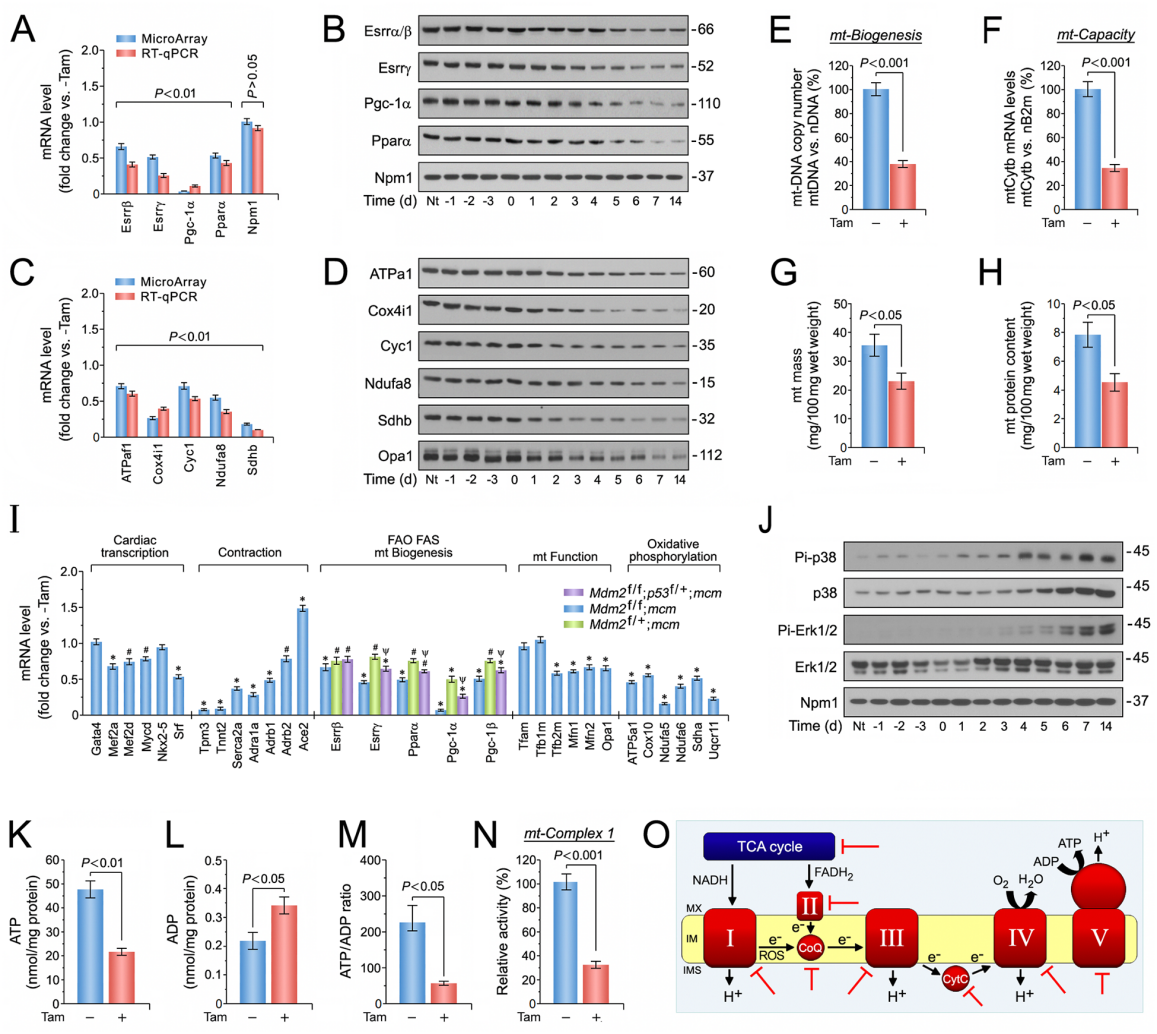
Mdm2 is involved in the maintenance of mitochondrial biogenesis and bioenergetics. (A) Expression levels of selected factors from top-ranked gene sets identified in Fig 4A that play a crucial role in the regulation of mitochondrial function and fatty acid oxidation as analyzed by RT-qPCR. *n* = 4. Left ventricular tissue samples from *Mdm2*^f/f^;*mcm* mice were analyzed at 7 days post-Tam. (B) Immunoblot analysis of key factors regulating mitochondrial biogenesis and fatty acid oxidation from *Mdm2*^f/f^;*mcm* mice. Western blots were repeated at least once with similar results. (C) Transcript levels of important constituents of the mitochondrial electron transport chain participating in oxidative phosphorylation in *Mdm2*^f/f^;*mcm* mice. *n* = 4. (D) Immunoblot analysis of important components of the mitochondrial electron transport chain complexes in *Mdm2*^f/f^;*mcm* mice. Western blots were repeated at least once with similar results. (E) Mitochondrial biogenesis, defined as relative DNA copy number of mitochondrial encoded Cytb gene normalized to the copy number of the nuclear gene B2m, was determined by qPCR in *Mdm2*^f/f^;*mcm* mice. *n* = 4. (F) Mitochondrial capacity, defined as relative mRNA levels of the nuclear gene cytochrome b (Cytb), a constituent of oxidative phosphorylation (OxPhos) complex III, normalized to B2m transcript expression, was determined by RT-qPCR in *Mdm2*^f/f^;*mcm* mice. (G) Decreased mitochondrial wet weight in ventricular tissue of *Mdm2*^f/f^;*mcm* mice post-Tam. *n* = 4. (H) Downregulation of total mitochondrial protein content in Mdm2-deficient ventricles. *n* = 4. (I) Cardiac-specific gene expression in the indicated Mdm2 and p53 mutant mice post-Tam as analyzed by RT-qPCR. *n* = 4. *P < 0.01 vs. -Tam. ^#^*P* < 0.05 vs. -Tam. Ψ < 0.05 vs. *Mdm2*^f/+^;*mcm* and *Mdm2*^f/f^;*mcm*. (J) Activation of p38 and Erk1/2 signal transduction pathways is associated with the development of decompensated cardiac hypertrophy in *Mdm2*^f/f^;*mcm* mice exposed to Tam. LV extracts were prepared at the indicated time points, and were immunoblotted with the antibodies as indicated on the left. The amount of phosphorylation was determined using phosphorylation site-specific antibodies to p38 and Erk1/2. Western blots were repeated at least once with similar results. (K) Markedly impaired ATP production indicates mitochondrial dysfunction in *Mdm2*^f/f^;*mcm* mice post-Tam. ATP levels were analyzed with a bioluminescence-based assay. *n* = 4. (L) Acute genetic deletion of Mdm2 evokes increases in ADP levels in *Mdm2*^f/f^;*mcm* mice post-Tam. *n* = 4. (M) Mdm2 is indispensable for the maintenance of proper energy metabolism as indicated by a significantly lower ATP/ADP ratio in *Mdm2*^f/f^;*mcm* mice post-Tam. *n* = 4. (N) Mitochondrial complex I activity in *Mdm2*^f/f^;*mcm* mice was determined with an ELISA assay employing isolated mitochondria and complex I-specific antibodies. The NADH dehydrogenase activity of complex I was determined colorimetrical by monitoring the oxidation of NADH to NAD+. *n* = 4. (O) Potential Mdm2-regulated genes in a well-established mt interaction network. Red block arrows indicate significantly downregulated genes in *Mdm2*^f/f^;*mcm* mice post-Tam. CoQ, coenzyme Q10. CytC, cytochrome c. IMM, inner mt membrane. IMS, inner membrane space. MMX, mt matrix. ROS, reactive oxygen species. (A to M) Tam- and vehicle-injected *Mdm2*^f/f^;*mcm* animals were investigated. Unless indicated otherwise left ventricular samples from 12-week-old mice were analyzed at 7 days post-Tam. Fig 6 data are means±s.e.m.

To determine whether activated p53, E2f1 and Myc in the absence of Mdm2 induces downregulation of major regulators of mitochondrial function and fatty acid oxidation, as predicted by our GSEA analysis (Fig 4*C*), we performed RT-qPCR and immunoblot analyses of left ventricular tissue samples from *Mdm2*^f/f^;*mcm* mice at 7 days post-Tam. Significant decreases in the mRNA levels of Esrrb/g Pgc-1a and Ppara/g in *Mdm2*^f/f^;*mcm* mice post-Tam were detected by RT-qPCR (*P* < 0.01) (Fig 6A), and confirmed by immunoblot analysis (Fig 6B).

The majority of the 1,500 mitochondrial proteins are encoded by nuclear genes. In contrast, the mitochondrial genome contains 13 protein-coding genes, 2 rRNAs and 22 tRNAs [91]. Since it has been firmly established that ATP levels are tightly correlated to contractility [92], we determined the expression levels of the top-ranked factors contained in the KEGG gene set oxidative phosphorylation (Fig 4C). Mitochondrial encoded genes all have the potential to modulate oxidative phosphorylation, albeit at differing levels, thus, it is crucial that mitochondrial transcription be coordinated with the other cellular signaling pathways that impose their own energy demands upon the cell [91,92]. Notably, we observed significant decreases in the expression of crucial components of respiratory chain complexes, including Atp5af1 (complex V, CV), Cox4i (CIV), somatic Cyc1 (CIII), Ndufa8 (CI) and Sdhb (CII), in RT-qPCR (*P* < 0.01) (Fig 6C) and Western blot analyses (Fig 6D) of left ventricular tissue samples derived from *Mdm2*^f/f^;*mcm* mice post-Tam. All these effects were not observed in vehicle-treated controls (Fig 6C).

Next, we investigated whether a global decline in mitochondrial biogenesis underlies the observed reduction in mitochondrial factors involved in OxPhos, because, for example, nuclear encoded proteins are actively transported into these organelles [91]. Therefore, mitochondrial DNA copy number was determined by qPCR of the mitochondrial gene cytochrome b (Cytb), normalized to levels of nuclear encoded *B2m* (beta-2 microglobulin). We noted a dramatically lower (-61%) mitochondrial copy number in *Mdm2*^f/f^;*mcm* post-Tam in comparison to untreated *Mdm2*^f/f^;*mcm* (*P* < 0.001) (Fig 6E). As expected from this result, mitochondrial respiratory capacity was also critically lower (-76%) in *Mdm2*^f/f^;*mcm* hearts post-Tam, while normal in controls (*P* < 0.001) (Fig 6F). We then analyzed the possibility, whether the observed decrease in mitochondrial Cytb transcription reflects a decrease in mitochondrial mass. Therefore, we directly measured mitochondrial wet weight and mitochondrial protein content in ventricular tissue of *Mdm2*^f/f^;*mcm* hearts post-Tam. Indeed, significant (*P* < 0.05) decreases in mitochondrial wet weight (Fig 6G) and mitochondrial protein content (Fig 6H) in *Mdm2*^f/f^;*mcm* exposed to Tam demonstrate that Mdm2 is involved in the maintenance of mitochondrial function.

We employed a RT-qPCR based screen to investigate transcriptional changes of gene sets that exert distinct function during cardiac hypertrophy. Intriguingly, we observed a significant downregulation (*P* < 0 .05 to 0.01) of key heart-specific transcription factors (Mef2a/d, Myocd, Srf) in Tam-treated *Mdm2*^f/f^;*mcm* when compared to control animals (Fig 6I). Expression of genes encoding important regulators of cardiac contractility (Adra1a, Adrb1, Serca2a, Ace2) were also significant lower in *Mdm2*^f/f^;*mcm* mice post-Tam (Fig 6I). Sarcomere organization profoundly influences cardiac function with abnormalities in this structure commonly observed in human HF. Strikingly, important contractile proteins (Myl2, Tpm3/4, Tnnt2) were downregulated in *Mdm2*^f/f^;*mcm* animals post-Tam (Fig 6I), demonstrating that Mdm2 is important for proper maintenance of the contractile apparatus. Beyond these genes, we also found key regulators of fatty acid metabolism (Esrrb/g, Ppara/g, Pgc-1a/b, Tfb1/2), mitochondrial fusion/fission (Mfn1/2, Opa1), and mitochondrial respiration (Atp5a1, Cox10, Ndufa5, Ndufa6, Sdha, Uqcr11) to be transcriptionally downregulated in Mdm2-mutant hearts (Fig 6I).

Next, we analyzed whether mRNA expression of Esrrb, Esrrg, Ppara, Pgc-1a, and Pgc-1b is subjected to regulation by p53, or occurs through Mdm2, independently of p53. Thus, RT-qPCR was carried out on total RNA isolated from Tam-treated *Mdm2*^f/+^;*mcm* and *Mdm2*^f/f^;*p53*^f/+^;*mcm* mice (Fig 6I). We observed, that transcript levels of Esrrg, Ppara and Pgc-1a were significantly higher in hearts of *Mdm2*^f/f^;*p53*^f/+^;*mcm* mice as compared to *Mdm2*^f/+^;*mcm* animals showing that these effects are regulated by p53, independent of Mdm2 (*P* < 0.05) (Fig 6I). Thus, it appears that Mdm2-dependent regulation of p53 transcriptional activity is necessary to maintain normal heart function.

It is well established, that the development of maladaptive cardiac hypertrophy involves the activation of distinct signal transduction pathways, including the mitogen-activated protein kinase (Mapk) signaling cascade [93]. Main Mapk signaling cascades consist of terminal effector kinases p38 and extracellular signal-regulated kinases (Erks). Both kinase families are each regulated by specific phosphorylation of adjacent Tyr and Thr recognition motifs [94]. Since hypertrophic growth of cardiomyocytes is associated with phosphorylation-dependent activation of p38 [95] and Erk1/2 [96], the phosphotransferase activity of these Mapks was investigated by immunoblotting employing antibodies that specifically recognize their phosphorylated active isoforms. Thus, we evaluated p38 activation by phosphorylation on Thr180/Tyr182, and found an increase in *Mdm2*^f/f^;*mcm* animals as early as 1d post-Tam (Fig 6J). Since the pro-hypertrophic function of Erk1/2 is associated with phosphorylation at Thr202/Tyr204, this Erk1/2 modification was analyzed by Western blotting employing phosphorylation site-specific antibodies. Notably, Tam-treatment triggered phosphorylation of Erk1/2 at Thr202/Tyr204 as early as day 4 in *Mdm2*^f/f^;*mcm* mice (Fig 6J). Taking these findings into consideration, we surmise that observed increases in p38 and Erk1/2 activation are a consequence of Mdm2 ablation. We also conclude that both signal transduction pathways are critical for the development of decompensated hypertrophy.

Mitochondrial dysfunction leads to lower production of mitochondrial ATP [97], and decreases in cytosolic ATP/ADP ratios are indicative of enhanced glycolytic ATP production, as recognized in heart failure. To gain a more complete understanding of the energy status in Mdm2-deficient cardiomyocytes, ADP levels were assessed. ATP levels were significantly lower (-54%) in the Mdm2-deficient hearts compared to vehicle controls (*P* < 0.001) (Fig 6K). We found higher ADP levels in the *Mdm2*^f/f^;*mcm* strain post-Tam (*P* < 0.05) in comparison to vehicle-injected controls (Fig 6L) and, thus, elevated ATP/ADP ratios (*P* < 0.05) (Fig 6M).

Immunocomplex assays confirmed a detrimental impact of Mdm2 on mitochondrial CI activity (-68%) in *Mdm2*^f/f^;*mcm* post-Tam, while such an effect was absent in vehicle-control animals (*P* < 0.001) (Fig 6N). This supports the hypothesis that negative regulation of crucial factors in the mitochondrial electron transport chain by inhibition of Pgc-1a, Ppara/g and Esrrb/g evokes severe mitochondrial deficiencies including fatty acid oxidation. Fig 6O illustrates the consequences of impaired Pgc-1a, Ppara/g and Esrrb/g axes on mitochondrial energy metabolism in the absence of Mdm2.

## Discussion

Here we report a role for the E3 ubiquitin ligase Mdm2 in differentiated cardiomyocytes where it supresses pathological cardiac growth through the promotion of p53 degradation. Mechanistically, we found that genetic ablation Mdm2 lead to increased levels of p53, in the absence of any acute stress. This effect sufficed to induce global alterations of the cardiac transcriptome and is reflective of ROS generation, apoptosis, fibrosis, broad mt deficiencies and hypertrophy in the Mdm2-deficient hearts. These processes culminated in the development of LV dysfunction and early death. This observation led us to realize that basal Mdm2 activity is indispensable for the control of proper heart function.

Oxidative stress plays an important role in the development of cardiac remodeling and HF. At higher levels, ROS causes direct contractile dysfunction by directly modifying proteins central to excitation-contraction coupling, and induces p53-mediated apoptosis [21,78–80]. ROS also stimulates proliferation of cardiac fibroblasts, and activates metalloproteinases leading to remodeling of the extracellular matrix [22]. Mdm2-deficient hearts develop early signs of ROS-induced oxidative damage. It appears reasonable to assume, therefore, that the majority of ROS derived from dysfunctional mt is due to p53 instigated changes in gene transcription. We have previously shown, that at low levels, p53 activity is an important regulator of normal heart function [98]. In the presence of acute stress, however, hyper-activated p53 induces predominantly apoptosis, as shown in this and other studies [37,38]. Thus, the second mechanism by which ablation of Mdm2 caused pathological cardiac hypertrophy is through the induction of p53-dependent apoptosis. The third, less characterized, pathway of maladaptive hypertrophy in the absence of Mdm2 probably involves downregulation of cardiac transcription factors, including Mef2a and Pgc-1a, key regulators of mt physiology. In a mouse model of telomere dysfunction, Sahin et al. demonstrated that telomeric dysfunction activated p53, which in turn can represses the Pgc-1a gene promoter causing HF [99]. Thus, the authors were the first who established a direct link between ROS-activated p53 and mt function.

It has been firmly established that p53 is spontaneously active in all tissues in mice with reduced Mdm2 expression [100,101]. Our previous analysis of a cardiac-specific p53 knockout mouse revealed that p53 regulates >1,000 differentially expressed transcripts in more than 20 gene sets relevant to cardiac architecture, excitation-contraction coupling, mt biogenesis and oxidative phosphorylation capacity [98]. This study defined a new role for p53 as a novel master regulator of the cardiac transcriptome, and suggests a central role for the Mdm2/p53 pathway in the maintenance of cardiac homeostasis that extends mechanistically far beyond the regulation of apoptosis and hypertrophy, as previously reported [46]. In fact, we are the first to report the fundamental importance of Mdm2 for the p53 pathway with respect to its strong impact on a wide spectrum of essential processes including cardiac gene transcription, ROS defence, and mt energy metabolism.

In the classical model, p53 is activated in mitotic cells by oncogenic stress, DNA damage and other stress signals, triggering apoptosis and proliferative arrest [35,37]. It has been well established that two classes of caspases, initiator caspases including caspase 8, and effector caspases, such as caspase 3, are instrumental in the execution of apoptosis [102]. In addition, two apoptotic signaling pathways have been identified. Caspase 8 controls the death receptor pathway whereas caspase 3 activation is dependent on mt cytochrome c release and caspase 9 function. However, independent studies have confirmed that caspase 8 is frequently activated in response to apoptotic signals in a death receptor independent manner, but is dependent on the participation of activated caspase 3 [103]. This might explain the co-activation of caspase 3/8 in Mdm2-deficient cardiomyocytes as observed in our study.

Our study provides genetic proof that p53 is spontaneously active in Mdm2-deficient hearts, corroborating the prevailing view, that inhibition of p53 is the primary function of Mdm2. An increase in p53 activity, in response to Mdm2 loss, triggered a phenotype that alters normal heart function. What is the role of p53 in the regulation of all these responses? Our analysis of various Mdm2- and p53-mutant mice demonstrates, that a rise in p53 levels is responsible for the initial transcriptional changes. However, it appears very likely that p53 is not the sole cause that drives the development of the cardiac phenotype in *Mdm2*^f/f^;*mcm* mice.

We observed that the pleiotropic transcription factors E2f1 [72] and Myc [73], both playing important roles in the initiation phase of cell death, are co-induced by p53 (Fig 4D and 4E). It has been shown previously that endogenous Myc is important for p53-induced apoptosis in response to DNA damage in the adult mouse intestine [104]. The proapoptotic activities of E2f1 and p53 are also co-regulated [105]. For example, p53 and E2f1 are stabilized and activated in response to various stresses through phosphorylation by ataxia telangiectasia mutated (ATM) [106,107] and the checkpoint kinases Chk1/2 [108,109]. E2f1 and p53 also cooperate [110] to transactivate many proapoptotic genes, including Bax, caspases, and cytochrome c [111,112]. Moreover, the expression of key apoptotic genes, including Apaf-1, Puma, and Siva, are reportedly co-regulated by both factors [10]. In isolated cardiomyocytes and adult mouse heart, ectopic overexpression of E2f1 evoked apoptosis [113–118].

It is also noteworthy that Mdm2 exerts p53-independent activities that are at least partially mediated by the E2f1 pathway [119]. For example, Mdm2 can physically interact with the retinoblastoma protein [120–122], E2F1 itself, and Dp1, a transcriptional co-activator of E2f1. All these interactions can stimulate transcriptional transactivation of E2f1 target genes [123,124].

Despite the negative regulation of p53 by Mdm2, physiological stress can induce low levels of p53 activity that is involved in the regulation of glycolysis, oxidative phosphorylation, and oxidative stress in mitotic cells [28, 98,125–128] and in the adult mouse heart [129,130]. Seminal studies have firmly established that p53-mediated apoptosis generates ROS [131–133]. Based on this work, several independent investigations revealed a primary role for p53 in the generation of oxidative stress and mt dysfunction in mouse models of HF [10,101].

In a recent study, we were able to demonstrate that Myc is a specific target of the E3 ubiquitin ligase Mule, in the adult mouse heart [10]. Loss of Mule, with activation of Myc, severely impacts redox homeostasis and mt function through negative regulation of Pgc-1a and Pink1, leading to oxidative stress, energy deprivation and cardiac dysfunction. Based on these findings, it appears highly likely that transcriptional activation of Myc by p53 participates in the development of HF in *Mdm2*^f/f^;*mcm* mice. The role of E2f1 in the generation of oxidative stress has remained unclear. However, ROS production is essential for the apoptotic function of E2f1 [134]. Surprisingly, E2f1 can repress mt respiration at baseline in mammalian cells [129], whereas loss of drosophila dE2f compromises mt function [135]. Cardiac ischemia-reperfusion injury in mice led to an activation of E2f1 [136]. In addition, infarct size was decreased by 40% in E2f1 knockout mice, as would be expected if E2f1 stimulated apoptosis.

Maladaptive cardiac hypertrophy can be characterized by activation of a fetal gene program [137–139]. This group of genes includes ANP, BNP, fetal isoforms of contractile proteins (bMHC, skeletal a-actin), and smooth muscle genes (a-actin, SM22a) [140,141]. While all these factors are highly expressed in fetal hearts, they are transcriptionally silenced in the early neonatal period. Their re-expression under conditions of pathological stress is considered to play a mechanistic key role development of decompensated cardiac hypertrophy [142]. Intriguingly, we observed that ANP, BNP, and bMHC transcript levels were downregulated at 14d post-Tam (Fig 3K). Reportedly, the ANP gene promoter contains DNA-recognition sequences and low affinity binding sites for Gata4/6, Mef2, Nkx2.5, serum response factor (Srf) and Tbx5, that alone or cooperatively regulate ANP expression in cardiomyocytes [138;143–145]. Moreover, The BNP promoter contains Gata4/6, Srf, nuclear factor of activated T-cells (Nfat), and thyroid hormone responsive DNA binding site, all of which participate in the activation of BNP gene transcription [145,146]. In fetal cardiomyocytes, and under pathological conditions in the adult heart, bMHC expression is directly regulated by Mef2, Nfat4/3c and Nkx2.5 [147–149]. The Gata4 transcription has previously been shown to transactivate the promoters of many cardiac genes, including ANP, BNP, and bMHC [150]. Also, Morin et al. demonstrated, that Mef2 physically interacts with Gata4, and these Mef2/Gata4 protein complexes are then recruited to target promoters that containing Gata4 but not Mef2-specific DNA interaction sites [151]. The physiological function of Mef2 in this cooperation is to potentiate the transcriptional activity of Gata4 [151]. Based on these findings, it appears that the decrease in ANP, BNP, and bMHC expression observed in our study is caused by a marked downregulation of Mef2 family members and Srf transcription factors (Fig 6I).

An important question to answer is what is p53’s role in the down-regulation of cardiac-specific gene transcription in *Mdm2*^f/f^;*mcm* mice? Work from our group has shown that cardiac-specific conditional co-deletion of p53 and Mdm2 (DKO) in adult mice led to downregulation of cardiac gene expression and the development of a dilated cardiomyopathy, in the absence of cardiac hypertrophy [77] with alterations in miRNA expression. In the present study, we describe a broad dysregulation in the control of cardiac-specific gene expression in Mdm2-deficient hearts, whereas the conditional ablation of p53 in adult mice provided a cardioprotective effect through the increased expression of Ca2+ handling and contractile proteins [77]. The transcriptomic signature in p53-deficient hearts not only comprised genes important for cardiac architecture and function, but also mt biogenesis and bioenergetics, glucose and fatty acid metabolism, and transcription factors including Esrrg, Gata4, Mef2a, Myod, Pgc-1a and Tfam. How can these disparate findings be reconciled? One plausible explanation for this relies on the supposition that the default position for the p53 network in the normal heart is “on” wherein p53 is active, broadly regulating the cardiac transcriptome, albeit at low levels. This is in contrast to the classical model, where p53 is “off” until induced by acute stress [37,38]. In this model, ‘hyperactive’ p53 is responsible for the induction of apoptosis and oxidative stress. Thus, we conclude from all these findings that the Mdm2/p53 and Myc/E2f1 pathways, with crosstalk between them, explains the observed phenotype in *Mdm2*^f/f^;*mcm* mice.

In post-mitotic cardiomyocytes, the auto-regulatory feedback loop between p53 and Mdm2 [152] appears to be important to maintain normal heart function by restraining aberrant p53 activation. However, the individual contribution of Mdm2 to accomplish this remains unclear [153]. Firstly, it is highly unlikely that p53 is the only substrate of Mdm2 in differentiated cardiomyocytes *in vivo* [34]. Experimental evidence also suggests that other E3 ligases can regulate p53 stability. Secondly, other endogenous p53 inhibitors including Mdm4, and the ubiquitin ligases Cop1, Huwe1 (Mule) [154], and Prh2 quench p53-dependent transcriptionally activation in proliferating cells, *in vitro* [35,36,155]. This can potentially influence the contribution of Mdm2 in p53 regulation. Intriguingly, cardiac-specific loss of Mule activated Myc and p53, leading to the development of a phenotype similar to the Mdm2-deficient mice. This current study provides genetic proof for the view that the extent to which Mdm2 participates in buffering p53’s activity is significant. Indeed, further studies are needed to ascertain whether reduced Mdm2 expression in human HF represents a novel genetic abnormality that predisposes patients to HF, or is instead a consequence of HF itself.

The protein kinase Akt plays an important role in the regulation of survival [156] and hypertrophic growth [157]. Various growth factors promote their effect through activation of the phosphatidylinositide-3-kinase (Pi3K) signaling cascade. Mdm2 is one of several Akt substrates whose activation mediates the anti-apoptotic activity of Akt. Independent studies demonstrated, that Pi3K signaling induces Akt-dependent phosphorylation and activation of Mdm2 that eventually inhibits p53-dependent apoptosis [44,156]. Since Mdm2 inhibits cardiac hypertrophy, whereas Akt promotes this process, the existence of a negative feedback loop has been postulated, enabling Mdm2 to restrain Akt action [46].

In our study, transcriptomic analysis of Mdm2-mutant hearts revealed inhibition of known Myc-and E2f-target genes that were activated by p53 and are crucial for the regulation of mt energy metabolism and ROS detoxification under conditions of cellular stress. This is consistent with studies demonstrating that Myc and E2f transcription factor complexes bound to target promoters that shift from trans-activating to trans-repressing [45,158]. The questions remain as to how are E2f1 and Myc activated in *Mdm2*^f/f^;*mcm* mice post-Tam, and how are the transcriptional activities of p53, E2f1 and Myc interconnected to evoke such a profound cardiac phenotype in these mice? Intriguingly, the p53/Mdm2 [159] and Rb/E2f1 [70] pathways are both defective in most human cancers, underlining the importance of both pathways in regulating apoptosis and proliferation. In addition, E2f1 has been shown to induce cardiomyocyte apoptosis [160]. Mechanistically, p53 and E2f1 are stabilized in response to DNA damage [70] by enhanced oxidative stress in the hearts of *Mdm2*^f/f^;*mcm* mice post-Tam.

We have previously shown that Pink1-deficient hearts exhibit decreased CI activity, directly leading to a drop in mt membrane potential [84]. Indeed, defective OxPhos through defects in complex 1 (CI) (via Pink1) and CIV (via Myc-mediated transrepression of Ndufa4) paired with sustained fatty acid oxidation (FAO) in the Mdm2-deficient myocardium signifies the uncoupling of FAO and OxPhos, which leads to ROS generation [104]. The coincident downregulation of important redox regulators (Pink1, Cat, Sirt3, Sod2), and antioxidants (Gpx4, Gsta1, Txnrd2) in a p53/E2f1/Myc-dependent manner, renders the Mdm2-deficient heart sufficiently incapable to mobilize a defence against ROS, thereby leading to DNA damage. Clearly, downregulation of Pgc-1a and Pink1 in the absence of Mdm2 represents the loss of a central homeostatic pillar supporting substrate utilization and ROS defense in the myocardium, which ultimately has a severe impact on cardiomyocyte function.

Work from our own lab has shown that Pink1-deficient hearts are hypertrophic and have higher degrees of cardiomyocyte apoptosis with fibrosis [84]. In addition, these mice have greater levels of oxidative stress as reflected by higher levels of lipid peroxidation, DNA damage, and decreased aconitase activity in conjunction with reduced activity of several cytoplasmic and mt antioxidative systems. In response to biomechanical stress, the Pink1 knockout mice developed a further exaggeration in the degree of cardiac hypertrophy. Recently, a link between Pink1 dysfunction and the electron transport chain was provided. Under physiological conditions, Pink1 maintains CI activity by phosphorylation of its subunit Ndufa10, critical for ubiquinone reduction through the holo-complex [161]. Ectopic expression of the phospho-mimetic mutant Ndufa10 rescued mt depolarization defects in Pink1 knockout MEFs. CI dysfunction was also reversed by expression of the phospho-mimetic Ndufa10 in cells from Parkinson’s patients carrying Pink1 mutations. Whether Ndufa10 phosphorylation is mediated directly by Pink1 or is an indirect effect of Pink1, remains to be determined. Pink1 loss constitutes a mt defect that establishes a pathological imbalance in ROS production, leading to derangements in mt bioenergetics [84]. With the generation of abnormal ROS levels within the mt, a cascade of events is initiated, including a decrease in MMP, activation of the mt permeability transition pore (mPTP), and efflux of protons causing a decline in mt bioenergetics. This supports the clinical relevance of our finding that protein levels of Pink1 in end-stage human HF were also diminished [84], supporting a reciprocal relationship between Pink1 activity and HF.

In this study, we identified several genes including Pgc-1a and Pink1, downregulated following Mdm2 ablation, as principle factors mediating p53/Myc/E2f1-mediated decreases in mt dysfunction and altered energy metabolism. Pgc-1a is a central effector of mt biogenesis and FAO through transcriptional co-activation of Ppara [162]. Previously, Pgc-1a expression was shown to be inversely regulated in Myc-induced cardiac hypertrophy in transgenic mice [163]. This finding closely mirrors the outcome of our study in which Myc overexpression, driven by p53/E2f1 activation, downregulates Pgc-1a and, consequently, decreases expression of the nuclear receptors Ppara and Esrrb/g. The combined activities of both transcription factor complexes oversee the principle mechanism of ATP production in the heart through transcriptional regulation of fatty acid uptake and FAO factors [164]. Thus, their absence in Mdm2-deficient heart leaves the heart vulnerable to impaired ATP production, which even takes on greater impact when the heart is stressed. Fig 6N illustrates the consequences of impaired Pgc-1a, Ppara/g and Esrrb/g axes on mt energy metabolism in the absence of Mdm2. In conclusion, our study provides a new level of understanding of how heart function is maintained at baseline, and how Mdm2 participates in the mediation of this process.

## Abbreviations

Ace2: angiotensin I converting enzyme (peptidyl-dipeptidase A) 2
Aco2: mitochondrial aconitase 2
Adra1a: adrenergic receptor, alpha 1a
Adrb2: adrenergic receptor, beta 2
aMHC: myosin, heavy polypeptide 6 cardiac muscle alpha (*Myh6*)
**ANP** (***Nppa***): natriuretic peptide type A
Apaf1: apoptotic peptidase activating factor 1
ATP5a1: ATP synthase, H+ transporting, mitochondrial F1 complex, alpha subunit 1
ATPaf1: ATP synthase mitochondrial F1 complex assembly factor 1
Bak: BCL2-antagonist/killer 1
Bax: BCL2-associated X protein
bMHC: myosin heavy polypeptide 7 cardiac muscle beta (*Myh7*)
**BNP** (***Nppb***): natriuretic peptide type B
Casp1: caspase 1
Casp3: caspase 3
Casp8: caspase 8
Cat: catalase
Col: collagen (for example: Col1a1, collagen type I alpha 1)
Cox10: cytochrome c oxidase assembly protein 10
Cox4i1: cytochrome c oxidase subunit IV isoform 1
Cx43: connexin 43 (*Gja1*, gap junction protein, alpha 1)
E2f1: E2F transcription factor 1
Esrrb: estrogen related receptor beta
Esrrg: estrogen related receptor gamma
Gata4: GATA binding protein 4
Gpx4: glutathione peroxidase 4
Gsta1: glutathione S-transferase, alpha 1
Lam: laminin alpha 1 (*Lama1*)
Mdm2: murine double-minute 2
Mef2a: myocyte enhancer factor 2A
Mef2d: myocyte enhancer factor 2D
Mfn1: mitofusin 1
Mfn2: mitofusin 2
Myc: myelocytomatosis oncogene
Myl3: myosin, light polypeptide 3
Mylk3: myosin light chain kinase 3
Ndufa5: NADH dehydrogenase (ubiquinone) 1 alpha subcomplex 5
Ndufa6: NADH dehydrogenase (ubiquinone) 1 alpha subcomplex 6 (B14)
Ndufa8: NADH dehydrogenase (ubiquinone) 1 alpha subcomplex 8
Nkx2-5: NK2 homeobox 5
Npm1: nucleophosmin 1
Opa1: optic atrophy 1
p53: transformation related protein 53 (*Trp53*)
Pgc-1a: peroxisome proliferative activated receptor gamma coactivator 1 alpha (*Ppargc1a*)
Pgc-1b: peroxisome proliferative activated receptor gamma coactivator 1 beta (*Ppargc1b*)
Pink1: PTEN induced putative kinase 1
Pln: phospholamban
Ppara: peroxisome proliferator activated receptor alpha
Ryr2: cardiac ryanodine receptor 2
Sdha: succinate dehydrogenase complex subunit A
Sdhb: succinate dehydrogenase complex subunit B
SERCA2: ATPase Ca^2+^ transporting cardiac muscle slow twitch 2 (*Atp2a2*)
Sirt3: sirtuin 3
Sod2: mitochondrial superoxide dismutase 2
Srf: serum response factor
Tcap: titin-cap
Tfam: transcription factor A, mitochondrial
Tfb1m: transcription factor B1, mitochondrial
Tfb2m: transcription factor B2, mitochondrial
Tnnc1: cardiac/slow skeletal troponin C
Tnni3: troponin I cardiac 3
Tnnt2: troponin T2, cardiac type
Tpm2: tropomyosin 2 beta
Tpm3: tropomyosin 3
Ttn: titan
Txnrd2: thioredoxin reductase 2
Uqcr11: ubiquinol-cytochrome c reductase, complex III subunit XI

All annotations use the mouse gene nomenclature. Designations in brackets refer to the official gene symbol.
